# Variation in Nordic Work-Related Cancer Risks after Adjustment for Alcohol and Tobacco

**DOI:** 10.3390/ijerph15122760

**Published:** 2018-12-06

**Authors:** Kristina Kjaerheim, Tor Haldorsen, Elsebeth Lynge, Jan Ivar Martinsen, Eero Pukkala, Elisabete Weiderpass, Tom K. Grimsrud

**Affiliations:** 1 Department of Research, Cancer Registry of Norway, N-0304 Oslo, Norway; tor.haldorsen0591@gmail.com (T.H.); jan.ivar.martinsen@kreftregisteret.no (J.I.M.); elisabete.weiderpass@kreftregisteret.no (E.W.); tom.k.grimsrud@kreftregisteret.no (T.K.G.); 2 Nykøbing Falster Hospital, University of Copenhagen, DK-4800 Nykøbing Falster, Denmark; elsebeth@sund.ku.dk; 3 Finnish Cancer Registry—Institute for Statistical and epidemiological Cancer Research, Unioninkatu 22, FI-00130 Helsinki, Finland; eero.pukkala@cancer.fi; 4 Faculty of Social Sciences, University of Tampere, 33014 Tampere, Finland; 5 Department of Community Medicine, Faculty of Health Sciences, University of Tromsø, The Arctic University of Norway, N-9037 Tromsø, Norway; 6 Department of Medical Epidemiology and Biostatistics, Karolinska Institutet, SE-171 77 Stockholm, Sweden; 7 Genetic Epidemiology Group, Folkhälsan Research Center and Faculty of Medicine, University of Helsinki, 00014 Helsinki, Finland

**Keywords:** occupation, cancer, confounding, alcohol, tobacco

## Abstract

*Background*: Alcohol and tobacco strongly increases the risk of cancers of the tongue, mouth, pharynx, larynx, and oesophagus, and are also established risk factors for cancer of the liver, colon, and rectum. It is well documented that these habits are unequally distributed among occupational groups. Most occupational cohort studies lack information on these potentially important confounders, and may therefore be prone to bias. *Aim*: The aim of the study is to present Nordic standardized incidence ratios (SIRs) for alcohol and tobacco related cancer by occupation, after adjustment for alcohol and tobacco, and to compare to the unadjusted SIRs. *Material and Methods*: The study is based on the Nordic Occupational Cancer (NOCCA) database. We used confirmatory factor analysis models for simultaneous analysis of the cancer sites related to alcohol and tobacco, to obtain factors that allow for computation of adjusted expected numbers from the reference rates. We then calculated adjusted SIRs for the relevant cancer sites for each occupation. *Results*: For some occupations and cancers, the changes of risk estimates were striking, from significantly high to significantly low and vice versa. Among Nordic farmers, unadjusted SIRs for cancer of the mouth and oesophagus were 0.56 (95% confidence interval (CI) 0.51–0.61) and 0.67 (CI 0.63–0.70), respectively. After adjustment, estimates changed to 1.10 (CI 1.01–1.21) and 1.16 (CI 1.10–1.22). Unadjusted SIR for pharynx cancer among wood workers was 0.83 (CI 0.75–0.91), adjusted SIR was 1.14 (CI 1.03–1.25). For larynx cancer, results in the opposite direction were seen: unadjusted SIR for economically inactive was 1.38 (CI 1.31–1.46) while the adjusted SIR was 0.91 (CI 0.86–0.96). *Conclusions*: Adjustment for the latent indicators of alcohol and tobacco consumption changed risk estimates for several occupations, gave a less confounded description of risk, and may guide in the identification of true risk factors.

## 1. Introduction

Alcohol and tobacco are strong risk factors, alone or in combination, for many types of cancer [1], and it has regularly been observed that these risk factors are unevenly distributed between occupations [2,3,4,5,6]. It is thus reasonable to expect that tobacco and alcohol may confound, more or less strongly, the association between occupation and cancer risk.

This potential confounding is a well-known problem in occupational cancer research, as in record linkage studies based on registry data computing standardized incidence or mortality ratios.

Several methods to account for this have been suggested and tested [7,8,9,10,11,12,13,14,15,16]. The availability of survey data on alcohol and tobacco habits allows for direct adjustments, while in indirect methods of adjustment one may select a reference group with presumed similar habits, rely on hypothetical distributions of alcohol and smoking habits, or use other smoking/alcohol related causes of death as comparison. Here, we applied a recently developed method for adjusting for alcohol and tobacco on a large occupational cohort of Nordic men and women. The aim was to assess the occupationally related risk of cancer at sites related to alcohol and tobacco consumption, after having adjusted for the effects of tobacco and alcohol.

## 2. Material and Methods

The study is based on the Nordic Occupational Cancer (NOCCA) database, previously described by Pukkala and co-workers [17]. Briefly, this database comprises a cohort of 7.4 million men and 7.5 million women from Denmark, Finland, Iceland, Norway, and Sweden identified from the national censuses performed between 1960 and 1990. Occupational information was coded according to the Nordic Occupational Classification (NYK) [18], which is a Nordic adaptation of the International Standard Classification of Occupations (ISCO) [19]. Codes were classified into 53 relatively specific occupational categories, and 1 additional category of economically inactive persons. Data was linked to the respective national cancer registries and follow-up for cancer was done until 2003 (Denmark and Norway), 2004 (Iceland), or 2005 (Finland and Sweden), accumulating 184.9 million and 199.5 million person-years for men and women, respectively.

The cancer sites included are those previously evaluated by the International Agency for Research on Cancer (IARC) to be associated to tobacco and alcohol consumption [1], i.e., cancers of the tongue, mouth, pharynx, larynx, oesophagus, liver, colon, and rectum. No data on consumption of alcohol and tobacco was available for all occupational groups in all five Nordic countries, and we used the incidence pattern of the relevant cancer types and confirmatory factor analysis models to estimate the effect on cancer risk ascribed to alcohol and tobacco. The method has been described in detail and tested by Haldorsen et al. [20] on a national cohort of Norwegian men from the 1970 census [21]. For an in-depth description of the methodology we refer to Haldorsen et al. [20]. 

Briefly, the pattern of risk at all cancer sites related both to alcohol and tobacco was considered to be the manifest (observed) expression of a latent (unobserved, common) variable for the combined effect of exposure, while a latent site specific (unique) factor allowed for the possible cancer site specific effect in each occupational group. The estimated factor loadings in the confirmatory factor analysis were then considered to represent the effect of exposure. From the final measurement models, predicted values for the common factor were computed by empirical Bayes’ means, and predicted values for the common factor and the estimated factor loadings were finally used to compute adjusted expected values. SIRs were computed separately for each country and gender, and finally combined into Nordic adjusted SIRs for each of the cancer sites.

## 3. Results

Results will be presented by cancer site for men and women, respectively, focusing on the direction and size of SIR changes with adjustment. Graphical illustrations of the changes in risk estimates are shown in Figure 1, Figure 2, Figure 3, Figure 4, Figure 5, Figure 6, Figure 7, Figure 8, Figure 9, Figure 10, Figure 11 and Figure 12, detailed results with SIRs and 95% confidence intervals are shown in Tables S1–S14.

### 3.1. Cancer of the Tongue, Mouth, and Pharynx in Men

**Figure 1:**
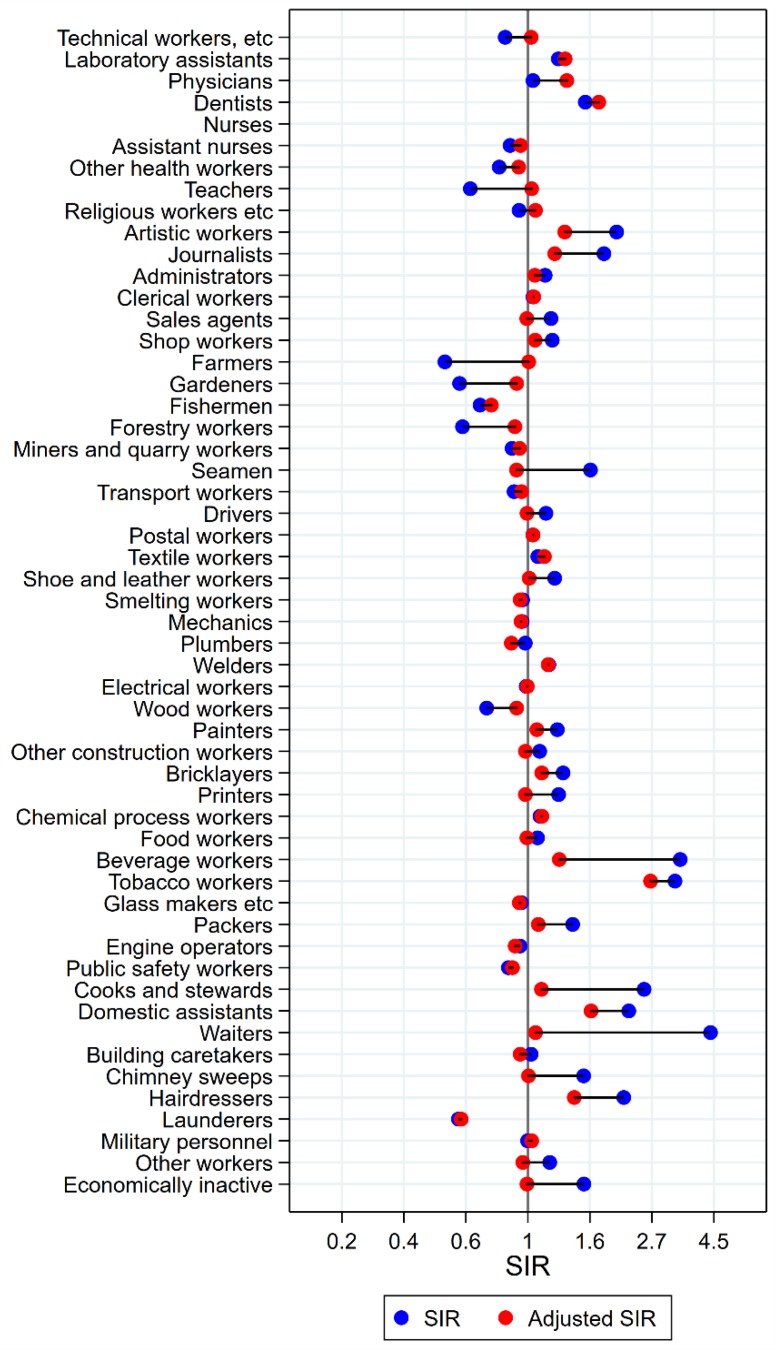
Unadjusted (blue) and alcohol and tobacco adjusted (red) SIRs for cancer of the tongue among 7,447,726 men in the Nordic countries, by occupation. Follow up 1961–2005.

Among the 16 groups with initial high SIRs of tongue cancer, only artistic workers remained at significantly elevated risk after adjustment for alcohol and tobacco (unadjusted SIR 2.05 (95% confidence interval (CI) 1.54–2.66) changed to SIR 1.35 (CI 1.02–2.75)) (Figure 1). Some of the adjustments were dramatic, from SIRs between 3 and 5 to less than 1.3 (waiters and beverage workers). The SIR observed among dentists (unadjusted SIR 1.59 (CI 0.91–2.58) increased to 1.77 (CI 1.01–2.88) with adjustment. No occupational group had significantly low risk of tongue cancer after adjustment for alcohol and tobacco. 

**Figure 2:**
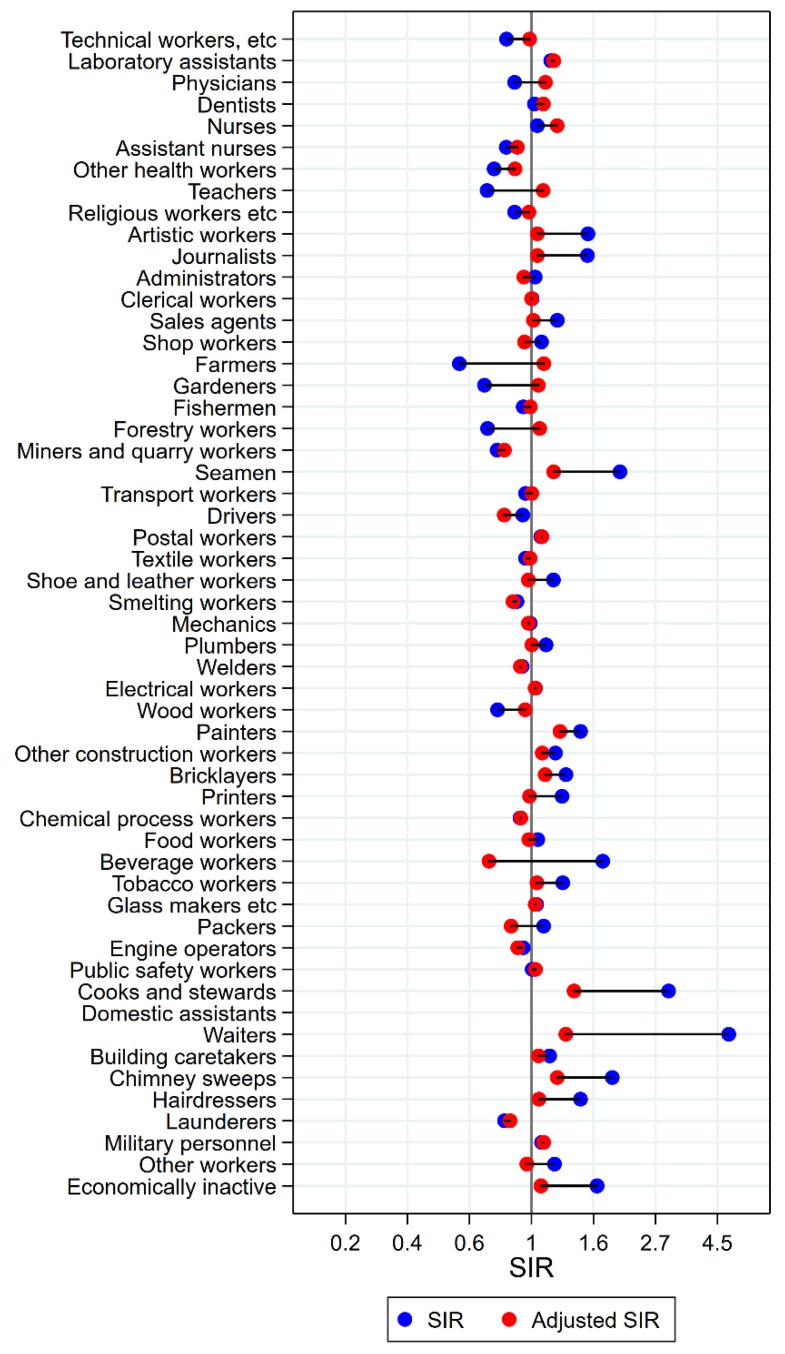
Unadjusted (blue) and alcohol and tobacco adjusted (red) SIRs for cancer of the mouth among 7,447,726 men in the Nordic countries, by occupation. Follow up 1961–2005.

The occupational groups of seamen, painters, cooks and stewards, and waiters all persistently had significantly elevated risk for cancer of the mouth after adjustment for alcohol and tobacco (Figure 2). Again, some groups showed dramatic changes from unadjusted SIRs between 3 and 5 to less than 1.5 (waiters and cooks and stewards). For artistic workers, journalists, sale agents, the combined group of other construction workers, bricklayers, printers, beverage workers, the group of other workers, and the economically inactive, the SIRs were no longer significantly elevated. In the opposite direction, the initial low risk among teachers, gardeners, forestry workers, and wood workers increased with adjustment, and was no longer different from the null value. For farmers, however, the unadjusted significantly low risk increased to a marginally significantly high risk with adjustment (SIR 0.56 (CI 0.51–0.61) changed to 1.10 (CI 1.01–1.21)). After adjustment, drivers and packers appeared with lowered risks (SIR 0.80 (CI 0.72–0.89) and 0.85 (CI 0.73–0.98), respectively).

**Figure 3:**
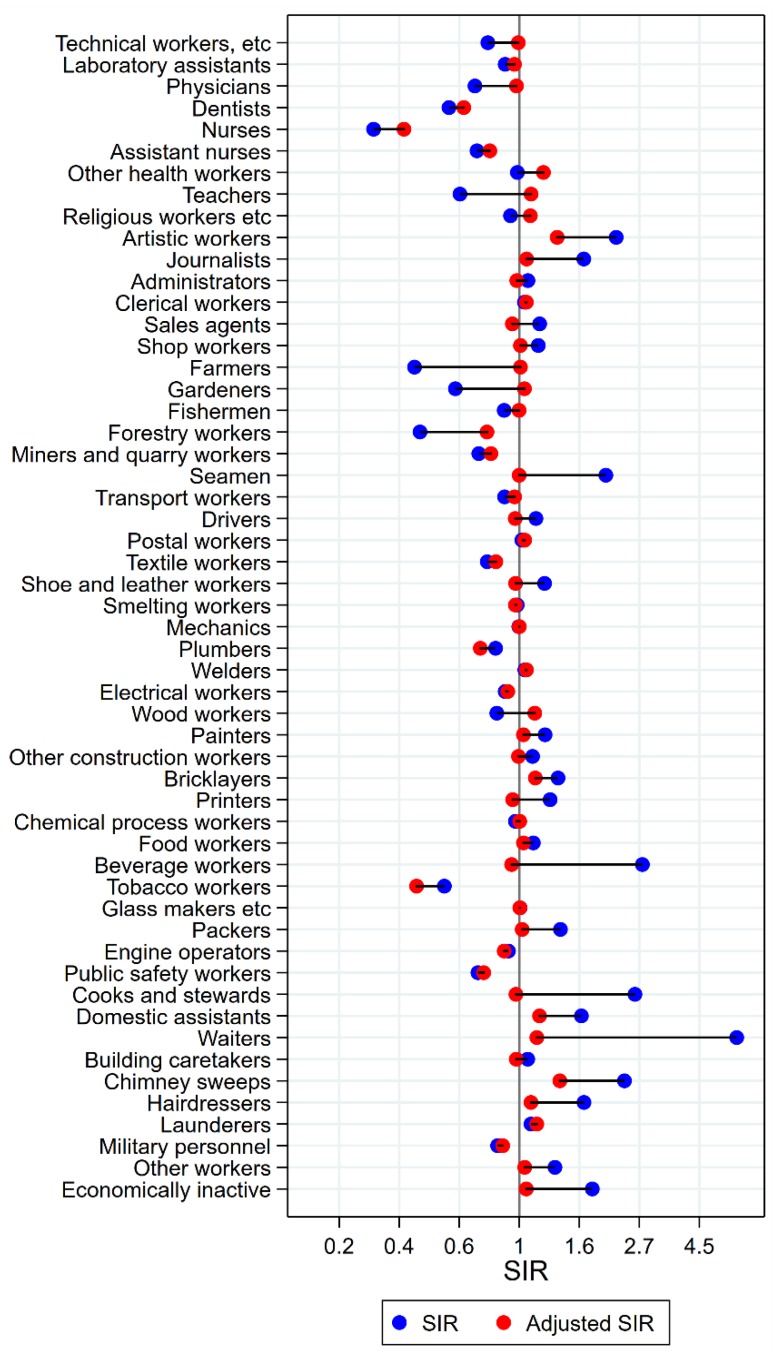
Unadjusted (blue) and alcohol and tobacco adjusted (red) SIRs for cancer of the pharynx among 7,447,726 men in the Nordic countries, by occupation. Follow up 1961–2005.

Of the 18 groups with significantly excess risk of pharyngeal cancer before adjustment, only artistic workers had persisting elevated risk after adjustment (SIR 2.24 (CI 1.85–2.69) changed to 1.37 (CI 1.13–1.64)) (Figure 3). In waiters, cooks and stewards, and beverage workers, unadjusted SIRs between 2 and 7 became practically normalised (SIRs between 0.94 and 1.16). Among forestry workers and public safety workers, risk remained low (adjusted SIRs of 0.76 (CI 0.61–0.95) and 0.74 (CI 0.60–0.91), respectively). For wood workers, a change in estimate from significantly low to significantly high was seen (SIR changed from 0.83 (CI 0.75–0.91) to 1.14 (CI 1.03–1.25)) with adjustment. 

### 3.2. Cancer of the Tongue, Mouth, and Pharynx in Women

**Figure 4:**
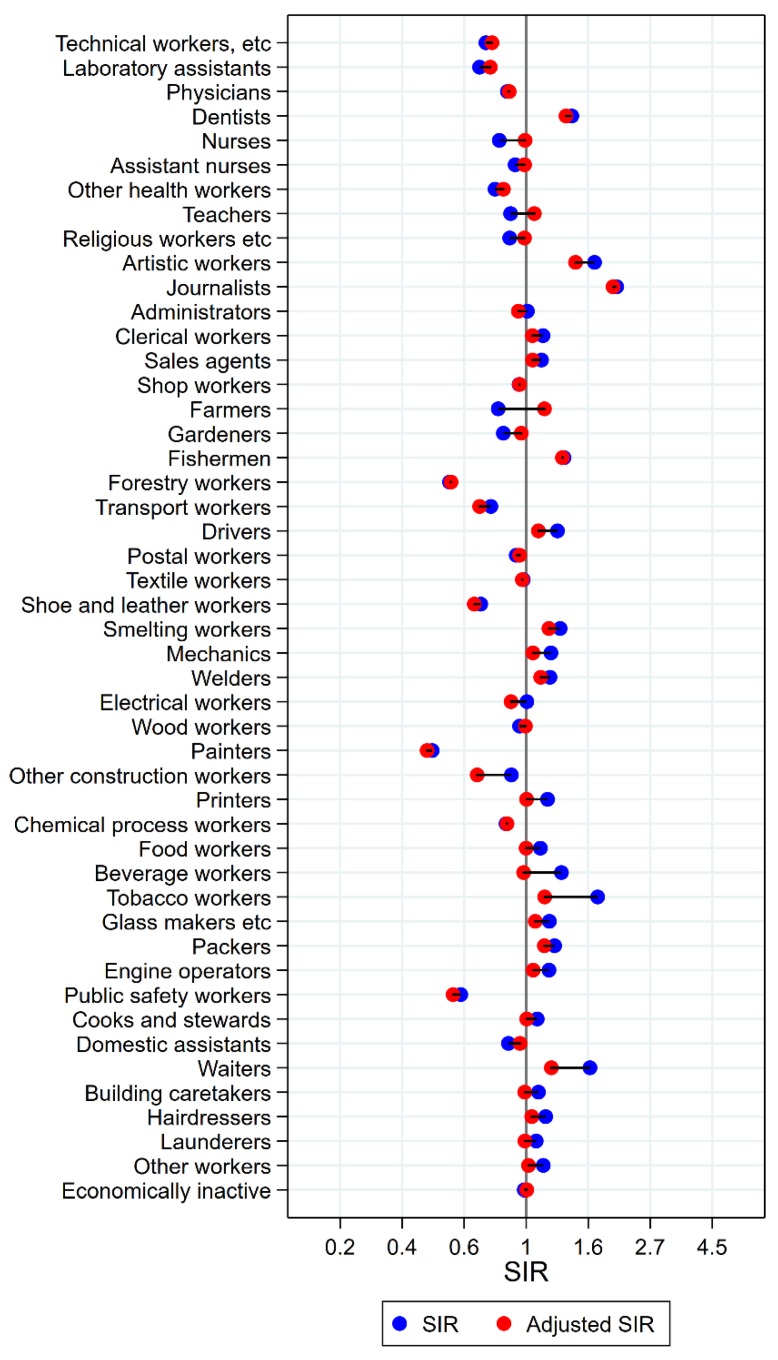
Unadjusted (blue) and alcohol and tobacco adjusted (red) SIRs for cancer of the tongue/mouth/pharynx among 7,454,847 women in the Nordic countries, by occupation. Follow up 1961–2005.

For women, combined analyses for cancers of the mouth, tongue, and pharynx were performed due to low numbers, to obtain statistical stability. For artistic workers, journalists, and waiters, risk estimates remained significantly elevated after adjustment (Figure 4). For artistic workers, SIR changed from 1.74 (CI 1.26–2.32) to 1.49 (1.09–1.99). Journalists had an unadjusted SIR of 2.07 (CI 1.31–3.11) changing to 2.01 (CI 1.28–3.02) after adjustment. Waiters’ SIR was lowered from 1.67 (CI 1.46–1.90) to 1.22 (CI 1.07–1.39). For clerical workers, tobacco workers, packers, and building caretakers the unadjusted significant elevations disappeared after adjustment for alcohol and tobacco. The significantly low SIRs among nurses, gardeners, and domestic assistants, on the other side, were not different from unity after adjustment. For female farmers, however, the significantly low SIR of 0.80 (CI 0.70–0.90) increased to 1.16 (1.02–1.31). For dentist, the risk was stably but non-significantly elevated (SIR 1.44 (CI 0.84–2.31) and 1.38 (CI 0.80–2.21)).

### 3.3. Laryngeal Cancer in Men

**Figure 5:**
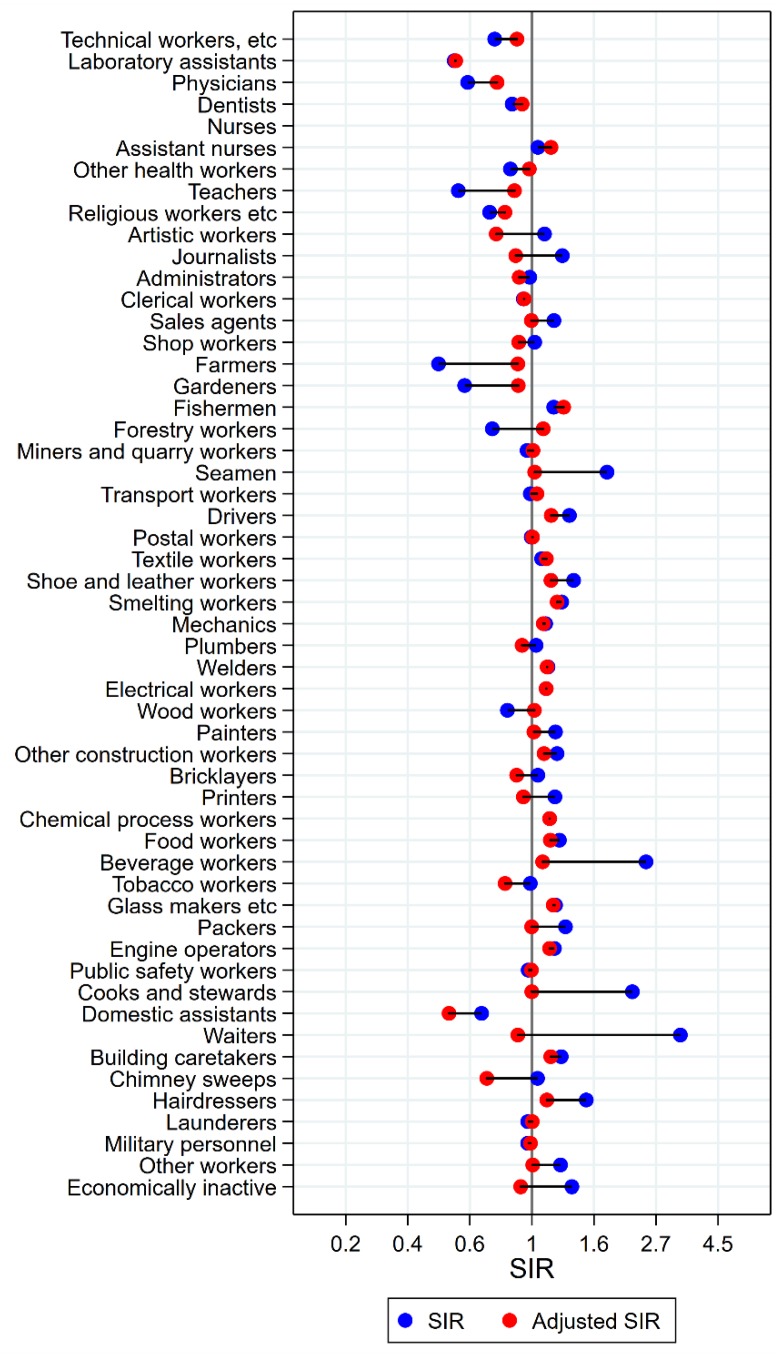
Unadjusted (blue) and alcohol and tobacco adjusted (red) SIRs for cancer of the larynx among 7,447,726 men in the Nordic countries, by occupation. Follow up 1961–2005.

Unadjusted, altogether 22 of the 53 + 1 occupational groups had significantly elevated SIRs for cancer of the larynx (Figure 5). After adjustment for alcohol and tobacco, only half of these had persisting elevations: fishermen, drivers, smelting workers, mechanics, electrical workers, other construction workers, chemical process workers, food workers, glass makers, engine operators, and building caretakers. Of the eight occupational groups with low unadjusted risk estimates, risk stayed significantly low for five: technical workers, laboratory assistants, teachers, the combined group of religious, juridical, and other academic workers, and farmers. Artistic workers and administrators had significantly low risk after adjustment.

### 3.4. Laryngeal Cancer in Women

**Figure 6:**
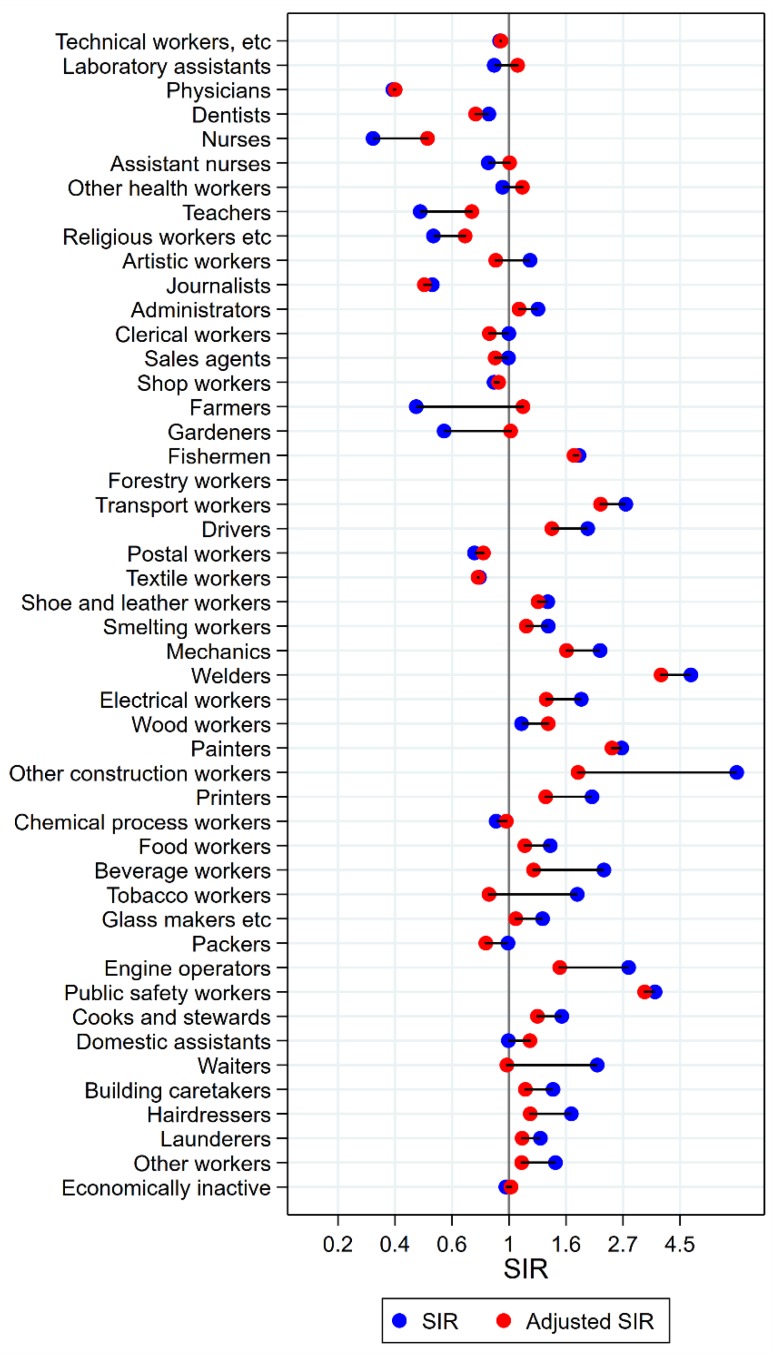
Unadjusted (blue) and alcohol and tobacco adjusted (red) SIRs for cancer of the larynx among 7,454,847 women in the Nordic countries, by occupation. Follow up 1961–2005.

Mechanics, public safety workers, and building caretakers remained at significantly elevated risk after adjustment (Figure 6). For electrical workers, printers, food workers, cooks and stewards, hairdressers, and other workers, risk was lowered to non-significant elevations between 12% and 38%. Among the groups with initial low SIRs, only nurses remained at significantly low risk after adjustment (SIR changed from 0.30 (CI 0.16–0.52) to 0.49 (CI 0.26–0.84) with adjustment), while for farmers and gardeners the adjusted risk was no longer different from unity. For clerical workers, the occupational group contributing the largest number of cases, a significantly low risk for larynx cancer appeared after adjustment (SIR 0.84, CI 0.74–0.95).

### 3.5. Oesophageal Cancer in Men

**Figure 7:**
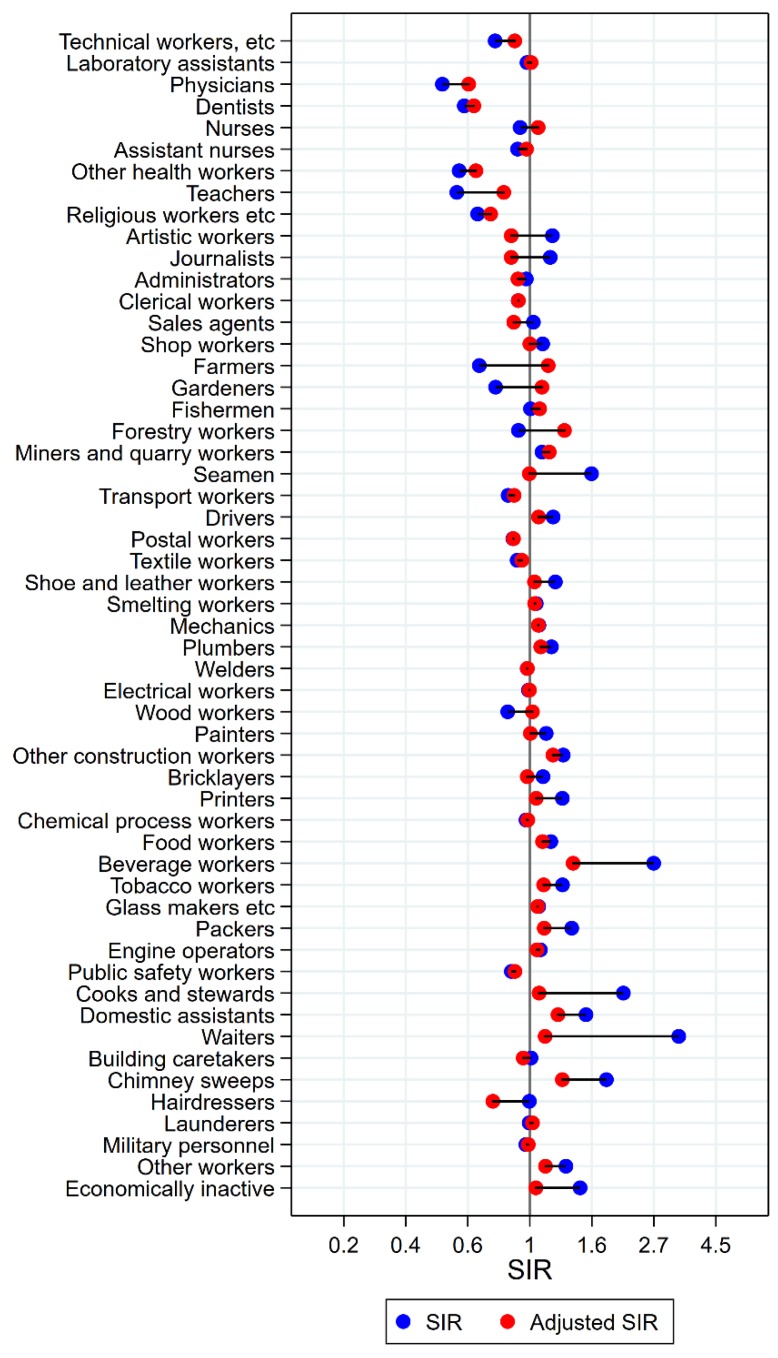
Unadjusted (blue) and alcohol and tobacco adjusted (red) SIRs for cancer of the esophagus among 7,447,726 men in the Nordic countries, by occupation. Follow up 1961–2005.

Consistently significantly high risk of oesophagus cancer was seen for drivers, mechanics, other constructions workers, food workers, beverage workers, packers, other workers, and those economically inactive (Figure 7). Consistently low risk, on the other hand, was seen for technical workers, physicians, dentists, other health workers, teachers, the group of religious, juridical, and other academic workers, clerical workers, and transport workers. Farmers and gardeners both changed profile from significantly low to significantly high risk after adjusting for alcohol and tobacco. Hairdressers had significantly low risk after adjustment (SIR 0.74 (CI 0.54–0.99)).

### 3.6. Oesophageal Cancer in Women 

**Figure 8:**
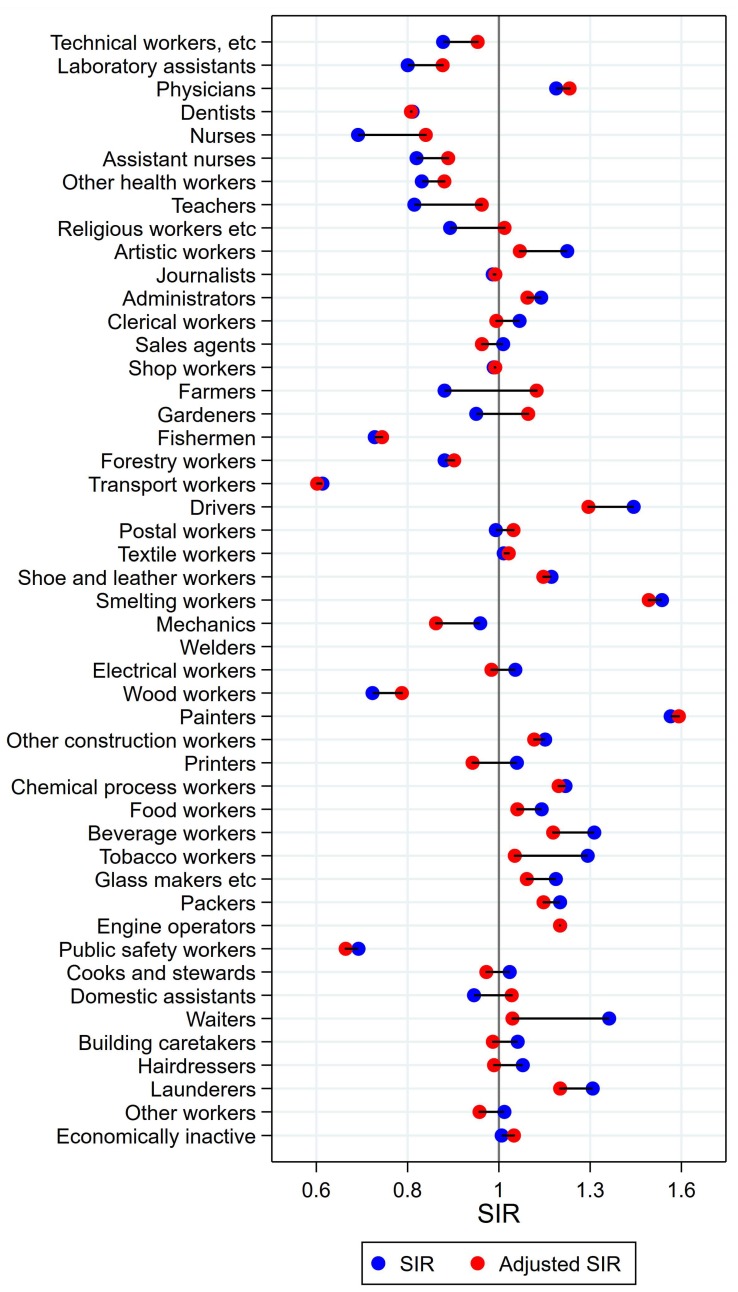
Unadjusted (blue) and alcohol and tobacco adjusted (red) SIRs for cancer of the esophagus among 7,454,847 women in the Nordic countries, by occupation. Follow up 1961–2005.

The unadjusted SIRs for oesophageal cancer showed quite small variation between occupations, and in general, risk estimates changed only moderately when adjusting for alcohol and tobacco (Figure 8). The unadjusted SIR for waiters was 1.35 (1.12–1.62), which was reduced to 1.04 (CI 0.84–1.24) after adjustment. Assistant nurses, teachers and farmers had significantly low risk before adjustment, which among the two former changed only moderately to non-significantly low. Among farmers, however, SIR was non-significantly above unity (SIR 1.11 (CI 0.96–1.27)) after adjustment.

### 3.7. Liver Cancer in Men

**Figure 9:**
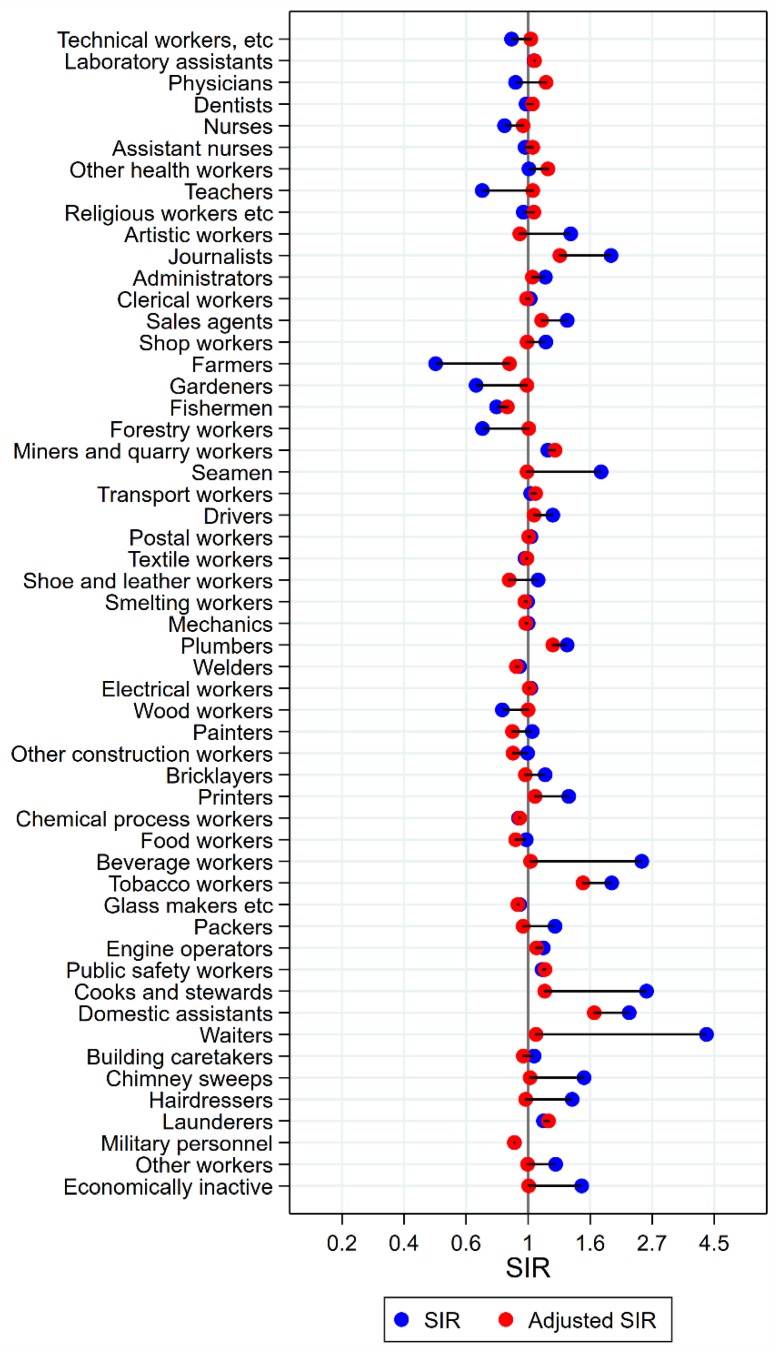
Unadjusted (blue) and alcohol and tobacco adjusted (red) SIRs for cancer of the liver among 7,447,726 men in the Nordic countries, by occupation. Follow up 1961–2005.

After adjustment, the elevated SIRs for liver cancer remained significantly high among journalists (1.29 (CI 1.01–1.62)), sales agents (1.12 (CI 1.05–1.19)), and plumbers (1.22 (CI 1.05–1.41)) (Figure 9). For miners and quarry workers and for public safety workers, risk increased after adjustment to significantly elevated SIRs of 1.24 (CI 1.01–1.51) and 1.15 (CI 1.01–1.29), respectively. Only two occupational groups had significantly low risk after adjustment for alcohol and tobacco; farmers with an SIR of 0.86 (CI 0.81–0.91) and other construction workers with SIR of 0.89 (CI 0.82–0.96).

### 3.8. Liver Cancer in Women

**Figure 10:**
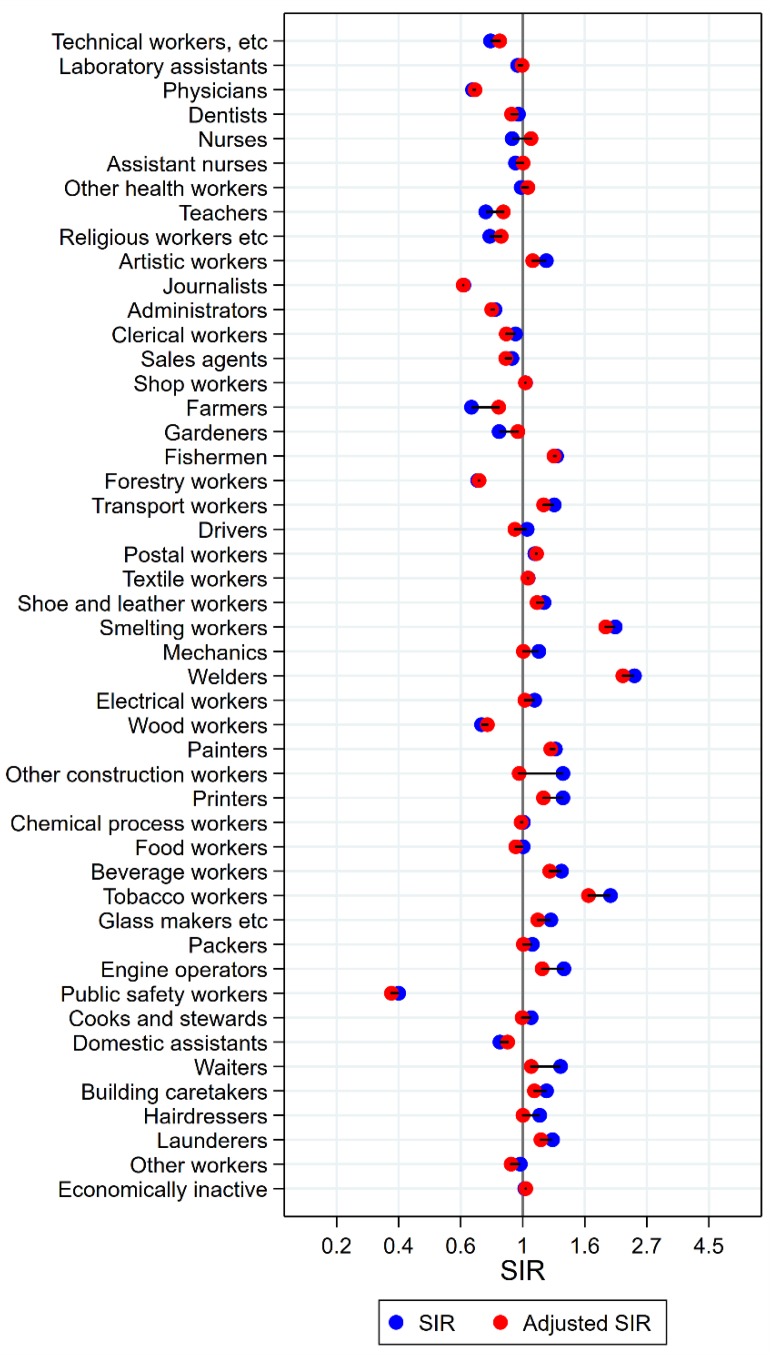
Unadjusted (blue) and alcohol and tobacco adjusted (red) SIRs for cancer of the liver among 7,454,847 women in the Nordic countries, by occupation. Follow up 1961–2005.

The unadjusted significantly elevated risks of liver cancer among smelting workers and building caretakers remained high after adjustment (SIRs changed from 2.11 (CI 1.09–3.68) to 1.95 (CI 1.01–3.41) and from 1.21 (CI 1.12–1.31) to 1.10 (CI 1.01–1.19), respectively) (Figure 10). The elevated risk among tobacco workers, waiters, and launderers disappeared after adjustment.

Teachers and farmers remained at a significantly low risk for liver cancer after adjustment for alcohol and tobacco, while for the group religious, juridical, and other academic workers, and among gardeners the SIRs increased to unity.

### 3.9. Colon Cancer in Men

**Figure 11:**
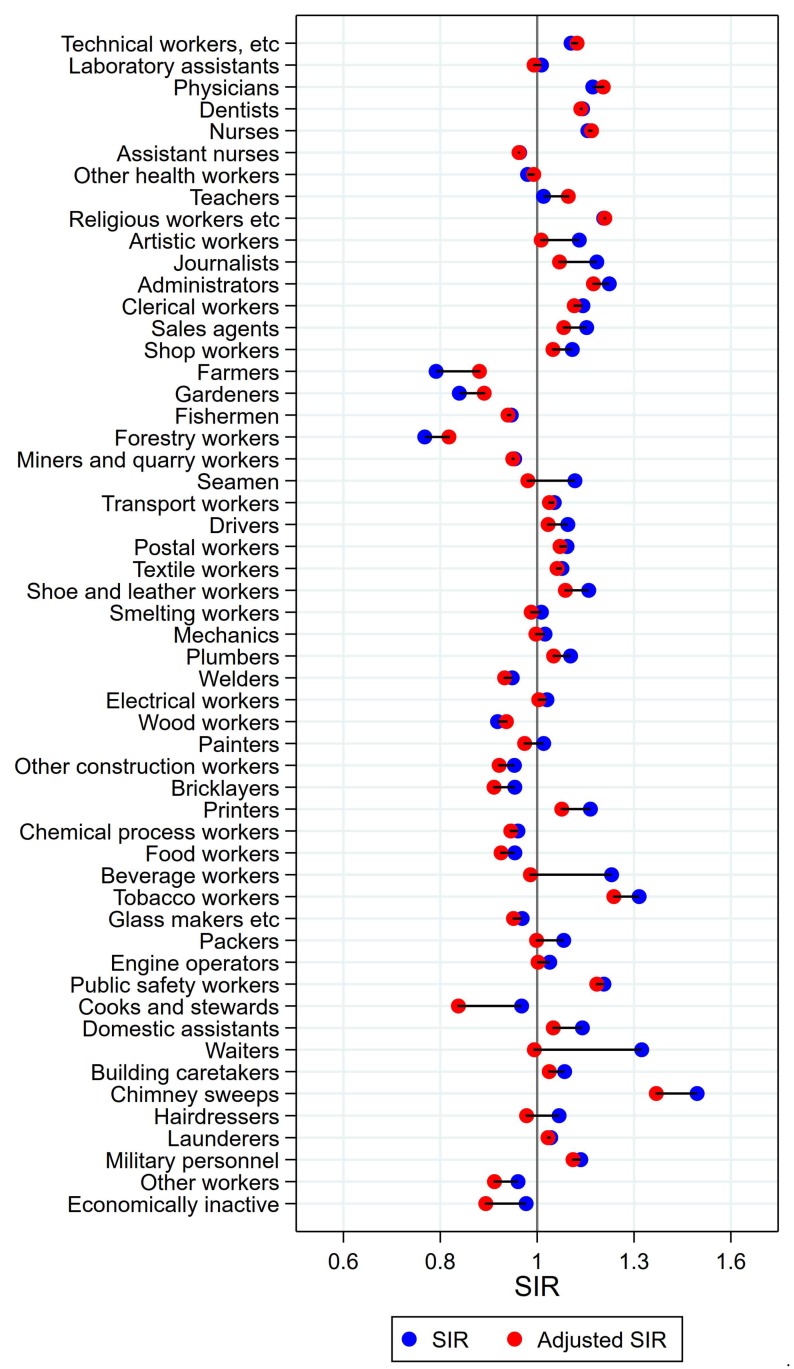
Unadjusted (blue) and alcohol and tobacco adjusted (red) SIRs for colon cancer among 7,447,726 men in the Nordic countries, by occupation. Follow up 1961–2005.

For most occupational groups, adjustment for alcohol and tobacco caused only relatively small changes in the risk estimates for colon cancer (Figure 11). For altogether 13 of the total 25 groups with initial significantly elevated risk, the high risk persisted after adjustment. These groups were technical workers, physicians, religious, juridical, and other academic workers, administrators, clerical workers, sales agents, shop workers, drivers, postal workers, printers, public safety workers, chimney sweeps, and military personnel. SIR increased to the level of significance in teachers (SIR 1.08 (CI 1.04–1.13)). Consistently low risk was seen among farmers, gardeners, fishermen, and forestry workers, as well as for wood workers, other construction workers, food workers, and for other workers. 

### 3.10. Colon Cancer in Women

Only minor changes appeared for colon cancer when adjusting for alcohol and tobacco (Table S12, not shown in graph). Risks remained significantly high for administrators, clerical workers, shop workers, postal workers, and textile workers. For printers, chemical process workers, glassmakers, waiters, and hairdressers the unadjusted significantly elevated risk estimates were only marginally reduced, but no longer significant, after adjustment.

### 3.11. Rectal Cancer in Men

**Figure 12:**
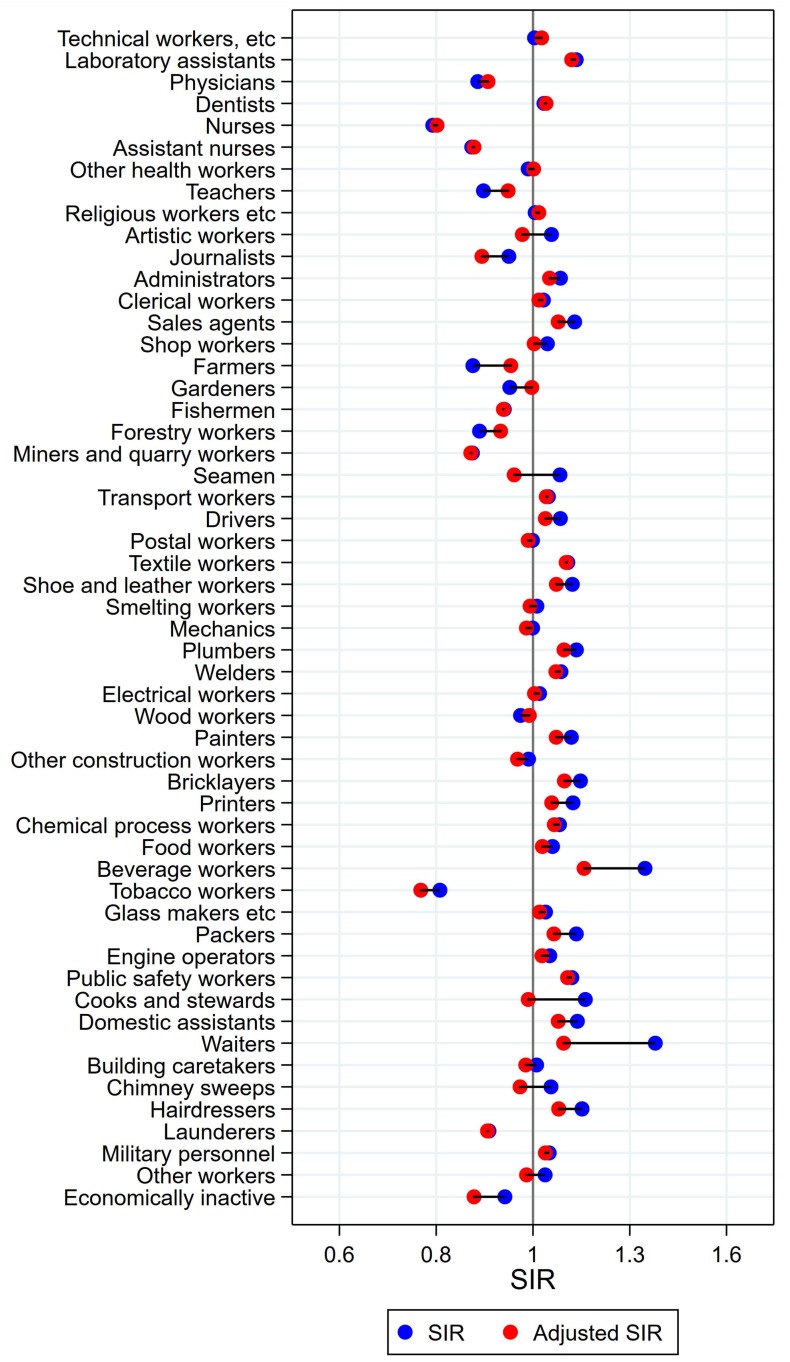
Unadjusted (blue) and alcohol and tobacco adjusted (red) SIRs for rectal cancer among 7,447,726 men in the Nordic countries, by occupation. Follow up 1961–2005.

For most occupational groups, risk of rectal cancer changed only marginally after adjustment for alcohol and tobacco (Figure 12). For administrators, sales agents, drivers, textile workers, plumbers, painters, bricklayers, packers, and public safety workers, the elevated risk of rectum cancer remained significantly elevated. Only waiters and beverage workers experienced a reduction in SIR of 0.20 or more. Teachers, farmers, fishermen, forestry workers, quarry workers, and the economically inactive group remained at significantly low risk for rectal cancer.

### 3.12. Rectal Cancer in Women

Results for rectum cancer were quite similar to those for colon cancer (Table S14, not shown in graph). The elevated risks among clerical workers, shop workers, and tobacco workers remained significantly high. For sales workers, textile workers, waiters, and other workers, SIRs were no longer significantly high. The significantly low SIRs among nurses, farmers and gardeners increased towards unity, and were no longer significant. Only for beverage workers, risk of rectum cancer remained significantly low after adjustment for alcohol and tobacco (SIRs 0.58 (CI 0.35–0.90) and 0.56 (CI 0.33–0.87), respectively).

## 4. Discussion

After adjustment, occupational variation in risk was in general decreased, as SIRs moved closer to the null value. This effect was, of course, an inevitable consequence of an approach that would take deviation from the mean to be a result of the factor for which we want to adjust. However, changes of risk estimates from significantly high to significantly low and vice versa were also seen. We note that overall, the effect of adjustment was strongest for cancers of the tongue, mouth, and pharynx. This was indeed expected, since the alcohol and tobacco related risks are considerably stronger for these sites than for cancer of the colon and rectum.

The NOCCA cohort, on which this study was based, is the largest occupational cohort ever assembled. Occupational categorization of the population was based on census information, independently from disease status. High quality cancer data from the cancer registries in the five Nordic countries were linked to each individual by use of the personal identification number [17]. To adjust for alcohol and tobacco consumption, we used the incidence rates of the relevant cancers to indirectly estimate exposure to alcohol and tobacco by use of confirmatory factor analysis. The tests for model fit fulfilled the suggested criterion [20]. The common factor in the model, representing exposure to alcohol and tobacco, was thus indirectly defined by the incidence of cancers related to alcohol and tobacco. There may however be heterogeneity in the historical smoking and drinking habits between occupational groups with respect to intensity (amount), duration in a lifetime perspective, and frequency—particularly in the balance between the tobacco and alcohol use—causing differences in the cancer pattern which may not be fully taken into account in the present model. The model allowed for variation between occupations that may be caused not only by occupation-specific exposure, but also by dietary factors, physical activity, or other factors related to socio-economic status. Such potential confounding must therefore be taken into account when interpreting the data. Due to the large data set and the multiple comparisons performed, it should also be noted that statistically significant SIRs do not necessarily imply scientifically interesting findings, and as always, results should be interpreted with caution for this reason.

Based on the unadjusted risk estimates for cancer associated with the combined effect of alcohol and tobacco and of tobacco alone, we have previously described three occupational clusters [22]. In men, the occupations which most consistently showed high risk of numerous cancer types, but specifically for cancers of the mouth, tongue, pharynx, and larynx, included waiters, cooks and stewards, beverage workers, seamen, and chimney sweeps (called the high-risk cluster). Two clusters of occupations with generally low cancer risks were seen in both men and women. The first one comprised farmers, gardeners, and forestry workers (the green low-risk cluster), the second one included groups with high education, specifically those in health and pedagogical work (the high education low-risk cluster). Below, results will be discussed in light of these clusters. Occupations which do not fall within any of the clusters, will also be pointed out, as needed.

**Figure 13:**
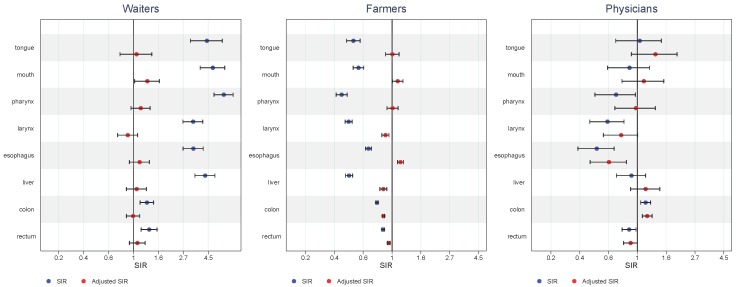
Unadjusted (blue) and adjusted (red) SIRs with 95% confidence intervals for alcohol and tobacco related cancer among male waiters, farmers, and physicians.

Typically, occupations within the high-risk cluster had few if any substantial risk elevations after adjustment (e.g., male waiters), while in the green low-risk cluster the initially observed low risk was closer to unity and sometimes even above (e.g., male farmers) (Figure 13). In the high education low-risk cluster, the low cancer risk also tended to move towards unity with adjustment for alcohol and tobacco.

### 4.1. The High-Risk Cluster

Among men, altogether 14 of the 53 + 1 included occupational groups had significantly elevated risk of 5 or more of the 8 included cancer sites before adjustment for alcohol and tobacco. These groups were artists, journalists, sales agents, shop workers, drivers, painters, printers, packers, beverage workers, cooks and stewards, waiters, hairdressers, the combined group of other workers, and the group of economically inactive. For these occupations, risk estimates after adjustment suggested that most, if not all, of the elevated risk may be ascribed to alcohol and tobacco consumption. For seamen, cooks and stewards, and waiters, only cancer of the mouth remained significantly elevated after adjustment. Estimates were however considerably reduced, and although other occupational or non-occupational risk factors cannot be excluded, specifically concerning seamen, the remaining risk may at least partly be caused by residual confounding from alcohol and tobacco. 

Among male artistic workers, risk of cancer of the tongue and pharynx was substantially reduced, but a significant 35–37% risk elevation was still present after adjustment. Among female artists, the persisting risk elevation was 49% for the combined group of cancer of the tongue/mouth/pharynx. Tarvainen et al. [23], using lung cancer incidence and liver cirrhosis mortality, respectively, to adjust for alcohol and tobacco, also found persistent risk elevations for cancer of the tongue and pharynx. The group artistic workers comprises authors (excluding journalists), sculptors, painters, painting restorers, composers, singers, musicians, dancers, and other types of artists. Among these, sculptors, painters, and painting restorers appear to be those with the highest potential for any carcinogenic exposure through their work. To clarify whether the persisting risk elevations are due to residual confounding by alcohol and/or tobacco or to strictly occupational exposure, more specific studies into these groups would be needed. 

The persisting risk of cancer of the mouth among male painters observed here is parallel to what was also seen in a case-control study by Richiardi et al. [24]. Painters are subject to a large number of potential carcinogens, and an association with cancer of the mouth is not implausible, although previous studies have more consistently pointed to a possible elevated risk of pharyngeal and oesophageal cancer in this group [25]. 

Among women, no proper high-risk cluster could be discerned [22]. Only waitresses had elevated risk on all 6 cancer sites included before adjustment. In this group, risk remained significantly high only for the combined group of tongue/mouth/pharynx cancer (SIR 1.22 (CI 1.07–1.39)) after adjustment, which is similar to what was seen among male waiters. For female journalists, risk of cancer of the tongue, mouth and pharynx remained unchanged after adjustment (adjusted SIR 2.01). It remains unclear which potential occupational or non-occupational exposures might cause this doubling in risk. Tarvainen et al. [23] also found that the risk of oral cancer remained significantly high after adjustment, and reported that liver cirrhosis mortality was exceptionally high among female journalists, while the lung cancer risk was only slightly elevated. This may indicate considerably higher alcohol consumption among female journalists than among the general female population, but relatively similar smoking habits. 

The occupational variation in cancer of the larynx was high, but among the occupations comprising the high-risk cluster, risk remained significantly high only in male drivers. Diesel exhaust has been suggested as a risk factor for laryngeal cancer [26], and elevated risk of laryngeal cancer has been suggested for lorry and van drivers [24], consistent with a role for diesel exhaust. In many occupations outside the high-risk cluster, however, risk of laryngeal cancer was persistently elevated, although with less than 30%, and most estimates were not essentially changed by adjustment for alcohol and tobacco. This was seen for fishermen, smelting workers, mechanics, electrical workers, other construction workers (including reinforced concreters, cement finishers, terrazzo workers, insulators, glaziers, underwater workers, and other unspecified building and construction workers), chemical process workers, food workers, glass makers, engine operators, and building caretakers. In addition to alcohol and tobacco, cancer of the larynx is considered causally related to exposure to asbestos and strong acid mists [27,28], and several studies suggest an association with silica, coal, and textile dust [29,30,31]. Our results demonstrate that more knowledge is needed regarding occupational causes of cancer of the larynx, as much of the variation on risk does not seem to be explained by the effect of alcohol and tobacco. 

Persistent risk elevation for cancer of the oesophagus was seen among male drivers and beverage workers, as well as among packers, the combined group of other workers, and the group of economically inactive. Occupational factors are not well established for oesophageal cancer, and there are no obvious common exposures among these groups to suggest an explanation to the elevated risk. In a study by Parent et al. [32], warehouse workers, food services workers, and workers from the miscellaneous food industry had suggested risk elevations for oesophageal cancer. While alcohol and smoking are considered the major risk factors specifically for squamous carcinoma of the oesoophagus, a diet low in fruit and vegetables and overweight and obesity are linked to increased risk of adenocarcinoma of the oesophagus in a dose dependent manner [33,34]. Several studies also find that physical activity reduces risk of cancer of the oesophagus [35], most consistently for adenocarcinoma, while an increasing sedentary lifestyle increases risk [34]. In the present study, we have not stratified by histological type, but it is plausible that a major part of the remaining risk may be attributed to risk factors relating to adenocarcinoma. 

Male journalists and sales agents had elevated risk of liver cancer after adjustment for the main risk factors alcohol and tobacco. Known occupational causes of liver cancer (vinyl chloride, 1,2-dichloropropane) do not seem relevant for these groups. Other risk factors, such as obesity and type II diabetes [36] seem more plausible, but no information is at present available to support or refute this hypothesis. 

For colon and rectum cancer, alcohol, tobacco, and the consumption of processed meat are established risk factors [37,38], while the evidence for an association with red meat is considered limited [38]. Physical inactivity and being overweight or obese also increases risk [39,40]. Except for asbestos, there are no known strictly occupational risk factors. In the high-risk cluster occupations in the present study, male printers, drivers, sales agents, packers, and painters had elevated risk of cancer of the colon and/or rectum after adjustment for alcohol and tobacco. Among these, especially drivers may have little physical activity at work. In a previous study in the NOCCA database, using a job-exposure-matrix, perceived physical strain at work was inversely associated with risk of colon cancer [41].

Postal workers, public safety workers, chimney sweeps, and military personnel are not included in either of the occupational clusters, but they had stable and significantly elevated risk for colon cancer after adjustment. Chimney sweeps and public safety workers (including firefighters, police officers, custom officers, guards, and guards) have adjusted colon cancer SIRs of 1.36 and 1.17, respectively. Elevated colon cancer risk has been observed in police officers [42] and among firefighters [43], but no definite etiological agent has been suggested. Several of these groups experience varied exposures from known and suspected carcinogens, and more specific studies would be required to elucidate potential associations with colon cancer risk.

### 4.2. The Green Low-Risk Cluster

This cluster comprises farmers, gardeners, and forestry workers. Unadjusted, all risk estimates for the included cancer sites were significantly low, except for oesophageal cancer among male forestry workers and female gardeners, for which the estimates were not different from unity. In farmers, the adjusted risk estimate for cancer of the mouth was significantly elevated by 10% among men and by 16% for the combined tongue/mouth pharynx cancer group among women. Relevant risk factors for cancer in the oral cavity in addition to alcohol and smoking are smokeless tobacco products, a poor diet with insufficient intake of fruit/vegetables, and infection with human papilloma virus (HPV). Although not implausible, we do not have data to support that male farmers actually use or have used more smokeless tobacco than other groups. If snuff use/pipe smoking were more strongly associated with cancer of the mouth than with tongue/pharynx cancer, such a habit might explain the excess risk of mouth cancer in male farmers, but it would not explain why risk is elevated also in female farmers. Engeland et al. [44] observed a dose-response relationship with pipe smoking for upper aerodigestive tract cancers, but results were not broken down by cancer site. Farmers are exposed to a variety of chemical, physical, and biological factors, and it is not unlikely that the elevated mouth cancer risk may be related to such.

After adjustment, all male occupational groups in this cluster showed 10–32% significantly elevated risk for cancer of the oesophagus. Among female farmers, risk of oesophageal cancer was non-significantly above unity (SIR 1.11 (CI 0.96–1.27)) after adjustment. An elevated risk of oesophageal cancer is a new finding, not easily explained by established risk factors such as overweight, obesity, or sedentary lifestyle. Previous studies on farmers’ cancer risk also have not typically observed elevated oesophageal cancer risk [45]. One earlier study found no association with pesticides use among farmers [46]. 

Risk of cancer of the liver, colon, and rectum changed only very moderately with adjustment for alcohol and tobacco in this low risk cluster, both among men and women, and remained for the most part significantly low. For cancer of the colon and rectum, the level of physical activity is assumed to contribute importantly to this finding. 

Wood workers have a similar risk profile as the described green low-risk cluster of occupations, with unadjusted SIRs being significantly low for cancers of the tongue, mouth, pharynx, liver, and colon. After adjustment, risk of pharyngeal cancer was however significantly elevated (SIR 1.14 (CI 1.03–1.25)). Wood dust is an established risk factor for cancer of the epipharynx, which has also been seen in a previous study which used a job exposure matrix in the analysis of the NOCCA data [47], and most probably explains the elevated risk among the wood workers. 

### 4.3. The High Education Low-Risk Cluster

This cluster includes groups with high education, specifically those in health and pedagogical work, technical workers (including engineers, physicists, architects, chemists, geologists, biologists, meteorologists, and related professionals), physicians, dentists, nurses, teachers, the group of religious, juridical, and other academic workers (including also archivists, librarians, economists, sociologists, psychologists and social work professionals), and administrators (including senior officials, managers and legislators working on behalf of governments, regional or local administrators, political parties, trade unions, and other organizations and enterprises). 

Among male dentist, the elevated risk of tongue cancer increased to a level of significance with adjustment, while among female dentist risk of cancer of the tongue/mouth/pharynx remained stable and non-significantly elevated (SIR 1.38 (CI 0.80–2.21)). By using another method of adjustment, Tarvainen et al. [23] also found an elevated tongue cancer risk in dentist in the same NOCCA data. An elevated risk in Swedish dentist was also described earlier (i.e., partly the same data material) [48]. No risk elevation was seen for any of the other alcohol and tobacco related sites, further suggesting that the tongue cancer risk seen among dentist may be caused by occupational exposure from chemical or biological sources. Data on cancer risk among dentists outside the Nordic countries was not found. In general, risk estimates for colon cancer in this cluster changed very little with adjustment for alcohol and tobacco, and risk remained high, especially among the men. Elevated colon cancer risk is commonly seen in groups with high socio-economic status, whether it be measured by education, income, occupation, or other indices. It is probable that low physical activity at work contributes to this elevated risk [39,40]. 

## 5. Conclusions

The normalisation of SIRs after adjustment in occupations groups known or suspected to have had a high consumption of alcohol and tobacco, indicated that our approach gave less biased estimates of cancer incidence. Similarly, support for a satisfactory adjustment was found in the change towards the null of SIR estimates in occupations with remarkably low unadjusted risks. The risk of pharynx cancer in wood workers changed from a significantly low SIR in men to an expected significantly elevated one, reinforcing the impression of a successful adjustment. In general, only small changes in the estimates appeared for cancer of the colon and rectum. Among remarkable findings, which deserve further attention, are the elevated risk of cancer of the mouth and oesophagus in farmers, cancer of the tongue in dentists, and the elevated risk of cancer of the larynx in a range of production/construction workers. In large datasets, our method of adjustment for alcohol and tobacco is useful for obtaining unbiased estimates of cancer risk in groups where no direct information on consumption is available.

Future research should be performed to corroborate the indicated knowledge gaps, and preventive efforts should be designed and targeted specifically at the occupations where cancer risk was appreciably reduced by adjustment for alcohol and tobacco.

## Supplementary Materials

The following are available online at http://www.mdpi.com/1660-4601/15/12/2760/s1, Table S1: Unadjusted and tobacco and alcohol adjusted SIRs for cancer of the tongue among 7,447,726 men in the Nordic countries, by occupation. Follow up 1961–2005, Table S2: Unadjusted and tobacco and alcohol adjusted SIRs for cancer of the mouth among 7,447,726 men in the Nordic countries, by occupation. Follow up 1961–2005, Table S3: Unadjusted and tobacco and alcohol adjusted SIRs for cancer of the pharynx among 7,447,726 men in the Nordic countries, by occupation. Follow up 1961–2005, Table S4: Unadjusted and tobacco and alcohol adjusted SIRs for cancer of the tongue/mouth/pharynx cancer among 7,454,847 women in the Nordic countries, by occupation. Follow up 1961–2005, Table S5: Unadjusted and tobacco and alcohol adjusted SIRs for cancer of the larynx among 7,447,726 men in the Nordic countries, by occupation. Follow up 1961–2005, Table S6: Unadjusted and tobacco and alcohol adjusted SIRs for cancer of the larynx among 7,454,847 women in the Nordic countries, by occupation. Follow up 1961–2005, Table S7: Unadjusted and tobacco and alcohol adjusted SIRs for cancer of the oesophagus among 7,447,726 men in the Nordic countries, by occupation. Follow up 1961–2005, Table S8: Unadjusted and tobacco and alcohol adjusted SIRs for cancer of the oesophagus among 7,454,847 women in the Nordic countries, by occupation. Follow up 1961–2005, Table S9: Unadjusted and tobacco and alcohol adjusted SIRs for liver cancer among 7,447,726 men in the Nordic countries, by occupation. Follow up 1961–2005, Table S10: Unadjusted and tobacco and alcohol adjusted SIRs for liver cancer among 7,454,847 women in the Nordic countries, by occupation. Follow up 1961–2005, Table S11: Unadjusted and tobacco and alcohol adjusted SIRs for colon cancer among 7,447,726 men in the Nordic countries, by occupation. Follow up 1961–2005, Table S12: Unadjusted and tobacco and alcohol adjusted SIRs for colon cancer among 7,454,847 women in the Nordic countries, by occupation. Follow up 1961–2005, Table S13: Unadjusted and tobacco and alcohol adjusted SIRs for rectal cancer among 7,447,726 men in the Nordic countries, by occupation. Follow up 1961–2005, Table S14: Unadjusted and tobacco and alcohol adjusted SIRs for rectal cancer among 7,454,847 women in the Nordic countries, by occupation. Follow up 1961–2005.
