# Supplementary material for: Variation in Nordic Work-Related Cancer Risks after Adjustment for Alcohol and Tobacco
Source: Int J Environ Res Public Health. 2018 Dec 6;15(12):2760. doi: 10.3390/ijerph15122760 (PMC6313809; doi:10.3390/ijerph15122760)
Supplement: Supplementary file 1 [file ijerph-15-02760-s001.pdf]

Table S1. Unadjusted and tobacco and alcohol adjusted SIRs for cancer of the tongue among 7,447,726 men in the Nordic countries, by occupation. Follow up 1961–2005.

			Unadjusted			Adjusted		
No	Occupational category	Obs	Exp	SIR	95% CI	Exp	SIR	95% CI
1	Technical workers, etc	336	403.53	**0.83**	**0.75-0.93**	327.26	1.03	0.92-1.14
2	Laboratory assistants	9	7.04	1.28	0.58-2.43	6.66	1.35	0.62-2.56
3	Physicians	27	25.91	1.04	0.69-1.52	19.73	1.37	0.90-1.99
4	Dentists	16	10.07	1.59	0.91-2.58	9.03	**1.77**	**1.01-2.88**
5	Nurses	0	1.85	0.00	0.00-2.00	1.53	0.00	0.00-2.42
6	Assistant nurses	6	6.93	0.87	0.32-1.89	6.36	0.94	0.35-2.05
7	"Other health workers"	15	18.90	0.79	0.44-1.31	16.15	0.93	0.52-1.53
8	Teachers	96	152.71	**0.63**	**0.51-0.77**	93.15	1.03	0.83-1.26
9	Religious workers etc	87	93.41	0.93	0.75-1.15	81.82	1.06	0.85-1.31
10	Artistic workers	56	27.38	**2.05**	**1.54-2.66**	41.61	**1.35**	**1.02-1.75**
11	Journalists	24	13.00	**1.85**	**1.18-2.75**	19.32	1.24	0.80-1.85
12	Administrators	278	241.90	**1.15**	**1.02-1.29**	262.78	1.06	0.94-1.19
13	Clerical workers	195	187.23	1.04	0.90-1.20	185.97	1.05	0.91-1.21
14	Sales agents	280	232.36	**1.21**	**1.07-1.36**	282.37	0.99	0.88-1.12
15	Shop workers	173	142.11	**1.22**	**1.04-1.41**	163.14	1.06	0.91-1.23
16	Farmers	283	552.83	**0.51**	**0.45-0.58**	281.09	1.01	0.89-1.13
17	Gardeners	82	142.19	**0.58**	**0.46-0.72**	89.77	0.91	0.73-1.13
18	Fishermen	40	58.83	**0.68**	**0.49-0.93**	53.70	0.74	0.53-1.01
19	Forestry workers	62	105.08	**0.59**	**0.45-0.76**	68.82	0.90	0.69-1.16
20	Miners and quarry workers	23	26.13	0.88	0.56-1.32	24.59	0.94	0.59-1.40
21	Seamen	108	65.18	**1.66**	**1.36-2.00**	118.34	0.91	0.75-1.10
22	Transport workers	81	90.55	0.89	0.71-1.11	85.30	0.95	0.75-1.18
23	Drivers	317	274.03	**1.16**	**1.03-1.29**	319.32	0.99	0.89-1.11
24	Postal workers	55	52.89	1.04	0.78-1.35	52.71	1.04	0.79-1.36
25	Textile workers	50	46.14	1.08	0.80-1.43	43.76	1.14	0.85-1.51
26	Shoe and leather workers	21	16.92	1.24	0.77-1.90	20.78	1.01	0.63-1.54
27	Smelting workers	78	81.40	0.96	0.76-1.20	83.03	0.94	0.74-1.17
28	Mechanics	368	385.60	0.95	0.86-1.06	388.80	0.95	0.85-1.05
29	Plumbers	46	47.00	0.98	0.72-1.31	52.59	0.87	0.64-1.17
30	Welders	58	48.99	1.18	0.90-1.53	49.25	1.18	0.89-1.52
31	Electrical workers	144	146.00	0.99	0.83-1.16	144.68	1.00	0.84-1.17
32	Wood workers	208	289.91	**0.72**	**0.62-0.82**	227.77	0.91	0.79-1.05
33	Painters	93	73.33	**1.27**	**1.02-1.55**	86.52	1.07	0.87-1.32
34	"Other construction workers"	174	158.23	1.10	0.94-1.28	177.51	0.98	0.84-1.14
35	Bricklayers	55	41.41	**1.33**	**1.00-1.73**	49.17	1.12	0.84-1.46
36	Printers	58	45.27	1.28	0.97-1.66	59.20	0.98	0.74-1.27
37	Chemical process workers	70	63.49	1.10	0.86-1.39	62.53	1.12	0.87-1.41
38	Food workers	88	81.43	1.08	0.87-1.33	88.69	0.99	0.80-1.22
39	Beverage workers	18	5.27	**3.42**	**2.02-5.40**	13.97	1.29	0.76-2.04
40	Tobacco workers	3	0.92	3.27	0.68-9.57	1.12	2.69	0.55-7.86
41	Glass makers etc	64	67.43	0.95	0.73-1.21	68.60	0.93	0.72-1.19
42	Packers	173	120.60	**1.43**	**1.23-1.67**	159.09	1.09	0.93-1.26
43	Engine operators	110	117.28	0.94	0.77-1.13	121.91	0.90	0.74-1.09
44	Public safety workers	63	73.73	0.85	0.66-1.09	71.35	0.88	0.68-1.13
45	Cooks and stewards	40	15.67	**2.55**	**1.82-3.48**	35.86	1.12	0.80-1.52
46	Domestic assistants	2	0.89	2.26	0.27-8.15	1.20	1.66	0.20-6.01
47	Waiters	41	9.40	**4.36**	**3.13-5.92**	38.56	1.06	0.76-1.44
48	Building caretakers	57	55.53	1.03	0.78-1.33	60.66	0.94	0.71-1.22
49	Chimney sweeps	6	3.83	1.57	0.58-3.41	5.97	1.00	0.37-2.19
50	Hairdressers	24	11.08	**2.17**	**1.39-3.22**	16.51	1.45	0.93-2.16
51	Launderers	4	7.01	0.57	0.16-1.46	6.85	0.58	0.16-1.50
52	Military personnel	44	44.10	1.00	0.72-1.34	42.68	1.03	0.75-1.38
53	"Other workers"	217	181.93	**1.19**	**1.04-1.36**	226.02	0.96	0.84-1.10
54	Economically inactive	401	255.19	**1.57**	**1.42-1.73**	403.88	0.99	0.90-1.10

Table S2. Unadjusted and tobacco and alcohol adjusted SIRs for cancer of the mouth among 7,447,726 men in the Nordic countries, by occupation. Follow up 1961–2005.

			Unadjusted			Adjusted		
No	Occupational category	Obs	Exp	SIR	95% CI	Exp	SIR	95% CI
1	Technical workers, etc	464	567.20	**0.82**	**0.75-0.90**	470.85	0.99	0.90-1.08
2	Laboratory assistants	11	9.42	1.17	0.58-2.09	9.19	1.20	0.60-2.14
3	Physicians	32	36.65	0.87	0.60-1.23	28.60	1.12	0.77-1.58
4	Dentists	15	14.65	1.02	0.57-1.69	13.63	1.10	0.62-1.82
5	Nurses	2	1.91	1.05	0.13-3.79	1.63	1.23	0.15-4.44
6	Assistant nurses	8	9.80	0.82	0.35-1.61	8.96	0.89	0.39-1.76
7	"Other health workers"	20	27.03	0.74	0.45-1.14	22.84	0.88	0.53-1.35
8	Teachers	149	213.12	**0.70**	**0.59-0.82**	135.68	1.10	0.93-1.29
9	Religious workers etc	109	124.86	0.87	0.72-1.05	111.28	0.98	0.80-1.18
10	Artistic workers	60	38.05	**1.58**	**1.20-2.03**	57.22	1.05	0.80-1.35
11	Journalists	28	17.83	**1.57**	**1.04-2.27**	26.69	1.05	0.70-1.52
12	Administrators	376	364.96	1.03	0.93-1.14	399.94	0.94	0.85-1.04
13	Clerical workers	275	273.39	1.01	0.89-1.13	274.73	1.00	0.89-1.13
14	Sales agents	400	324.80	**1.23**	**1.11-1.36**	393.82	1.02	0.92-1.12
15	Shop workers	250	230.53	1.08	0.95-1.23	264.48	0.95	0.83-1.07
16	Farmers	469	838.70	**0.56**	**0.51-0.61**	424.66	**1.10**	**1.01-1.21**
17	Gardeners	146	213.26	**0.68**	**0.58-0.81**	138.08	1.06	0.89-1.24
18	Fishermen	84	89.77	0.94	0.75-1.16	84.77	0.99	0.79-1.23
19	Forestry workers	104	148.13	**0.70**	**0.57-0.85**	97.34	1.07	0.87-1.29
20	Miners and quarry workers	28	36.94	0.76	0.50-1.10	34.85	0.80	0.53-1.16
21	Seamen	191	93.54	**2.04**	**1.76-2.35**	159.77	**1.20**	**1.03-1.38**
22	Transport workers	126	132.16	0.95	0.79-1.14	125.72	1.00	0.83-1.19
23	Drivers	363	389.23	0.93	0.84-1.03	452.34	**0.80**	**0.72-0.89**
24	Postal workers	82	76.05	1.08	0.86-1.34	75.37	1.09	0.87-1.35
25	Textile workers	68	71.35	0.95	0.74-1.21	68.76	0.99	0.77-1.25
26	Shoe and leather workers	31	25.97	1.19	0.81-1.69	31.78	0.98	0.66-1.38
27	Smelting workers	112	125.92	0.89	0.73-1.07	130.00	0.86	0.71-1.04
28	Mechanics	544	549.37	0.99	0.91-1.08	557.48	0.98	0.90-1.06
29	Plumbers	73	64.89	1.13	0.88-1.41	72.77	1.00	0.79-1.26
30	Welders	59	63.63	0.93	0.71-1.20	64.49	0.91	0.70-1.18
31	Electrical workers	206	199.38	1.03	0.90-1.18	199.99	1.03	0.89-1.18
32	Wood workers	319	419.37	**0.76**	**0.68-0.85**	335.73	0.95	0.85-1.06
33	Painters	160	107.71	**1.49**	**1.26-1.73**	127.07	**1.26**	**1.07-1.47**
34	"Other construction workers"	289	238.23	**1.21**	**1.08-1.36**	264.90	1.09	0.97-1.22
35	Bricklayers	86	65.11	**1.32**	**1.06-1.63**	77.04	1.12	0.89-1.38
36	Printers	84	65.61	**1.28**	**1.02-1.59**	85.27	0.99	0.79-1.22
37	Chemical process workers	85	93.24	0.91	0.73-1.13	92.65	0.92	0.73-1.13
38	Food workers	135	128.30	1.05	0.88-1.25	137.89	0.98	0.82-1.16
39	Beverage workers	16	9.00	**1.78**	**1.02-2.89**	22.52	0.71	0.41-1.15
40	Tobacco workers	2	1.55	1.29	0.16-4.65	1.91	1.04	0.13-3.77
41	Glass makers etc	105	100.73	1.04	0.85-1.26	101.92	1.03	0.84-1.25
42	Packers	193	174.92	1.10	0.95-1.27	227.36	**0.85**	**0.73-0.98**
43	Engine operators	150	160.38	0.94	0.79-1.10	167.58	0.90	0.76-1.05
44	Public safety workers	104	103.42	1.01	0.82-1.22	100.71	1.03	0.84-1.25
45	Cooks and stewards	64	21.18	**3.02**	**2.33-3.86**	45.39	**1.41**	**1.09-1.80**
46	Domestic assistants	0	0.92	0.00	0.00-4.01	1.23	0.00	0.00-3.00
47	Waiters	67	13.64	**4.91**	**3.81-6.24**	50.77	**1.32**	**1.02-1.68**
48	Building caretakers	93	80.32	1.16	0.93-1.42	87.79	1.06	0.85-1.30
49	Chimney sweeps	10	5.21	1.92	0.92-3.53	8.12	1.23	0.59-2.27
50	Hairdressers	27	18.18	1.49	0.98-2.16	25.41	1.06	0.70-1.55
51	Launderers	9	11.18	0.80	0.37-1.53	10.69	0.84	0.38-1.60
52	Military personnel	68	62.65	1.09	0.84-1.38	61.67	1.10	0.86-1.40
53	"Other workers"	339	281.55	**1.20**	**1.08-1.34**	351.88	0.96	0.86-1.07
54	Economically inactive	596	351.10	**1.70**	**1.56-1.84**	552.24	1.08	0.99-1.17

Table S3. Unadjusted and tobacco and alcohol adjusted SIRs for cancer of the pharynx among 7,447,726 men in the Nordic countries, by occupation. Follow up 1961–2005.

			Unadjusted			Adjusted		
No	Occupational category	Obs	Exp	SIR	95% CI	Exp	SIR	95% CI
1	Technical workers, etc	601	780.88	**0.77**	**0.71-0.83**	606.00	0.99	0.91-1.07
2	Laboratory assistants	11	12.38	0.89	0.44-1.59	11.46	0.96	0.48-1.72
3	Physicians	34	49.19	**0.69**	**0.48-0.97**	34.82	0.98	0.68-1.36
4	Dentists	11	19.76	0.56	0.28-1.00	17.47	0.63	0.31-1.13
5	Nurses	1	3.37	0.30	0.01-1.66	2.61	0.38	0.01-2.13
6	Assistant nurses	10	14.22	0.70	0.34-1.29	12.78	0.78	0.38-1.44
7	"Other health workers"	36	36.59	0.98	0.69-1.36	29.45	1.22	0.86-1.69
8	Teachers	175	286.85	**0.61**	**0.52-0.71**	158.69	1.10	0.95-1.28
9	Religious workers etc	164	176.43	0.93	0.79-1.08	149.58	1.10	0.93-1.28
10	Artistic workers	116	51.75	**2.24**	**1.85-2.69**	84.60	**1.37**	**1.13-1.64**
11	Journalists	42	24.55	**1.71**	**1.23-2.31**	39.50	1.06	0.77-1.44
12	Administrators	498	463.61	1.07	0.98-1.17	508.61	0.98	0.89-1.07
13	Clerical workers	379	363.03	1.04	0.94-1.15	357.41	1.06	0.96-1.17
14	Sales agents	523	441.64	**1.18**	**1.08-1.29**	554.21	0.94	0.86-1.03
15	Shop workers	341	291.13	**1.17**	**1.05-1.30**	337.82	1.01	0.90-1.12
16	Farmers	435	1041.33	**0.42**	**0.38-0.46**	430.89	1.01	0.92-1.11
17	Gardeners	161	274.25	**0.59**	**0.50-0.69**	154.24	1.04	0.89-1.22
18	Fishermen	93	105.52	0.88	0.71-1.08	93.18	1.00	0.81-1.22
19	Forestry workers	84	192.09	**0.44**	**0.35-0.54**	109.98	**0.76**	**0.61-0.95**
20	Miners and quarry workers	35	49.05	**0.71**	**0.50-0.99**	44.33	0.79	0.55-1.10
21	Seamen	241	117.23	**2.06**	**1.80-2.33**	241.31	1.00	0.88-1.13
22	Transport workers	152	171.87	0.88	0.75-1.04	158.14	0.96	0.81-1.13
23	Drivers	590	513.55	**1.15**	**1.06-1.25**	610.33	0.97	0.89-1.05
24	Postal workers	104	101.81	1.02	0.83-1.24	99.44	1.05	0.85-1.27
25	Textile workers	70	91.46	**0.77**	**0.60-0.97**	85.09	0.82	0.64-1.04
26	Shoe and leather workers	41	33.23	1.23	0.89-1.67	42.30	0.97	0.70-1.31
27	Smelting workers	162	164.85	0.98	0.84-1.15	167.40	0.97	0.82-1.13
28	Mechanics	739	742.14	1.00	0.93-1.07	738.83	1.00	0.93-1.08
29	Plumbers	72	87.75	0.82	0.64-1.03	99.64	**0.72**	**0.57-0.91**
30	Welders	93	88.79	1.05	0.85-1.28	87.62	1.06	0.86-1.30
31	Electrical workers	242	272.37	0.89	0.78-1.01	266.39	0.91	0.80-1.03
32	Wood workers	450	542.64	**0.83**	**0.75-0.91**	395.60	**1.14**	**1.03-1.25**
33	Painters	176	142.04	**1.24**	**1.06-1.44**	170.07	1.03	0.89-1.20
34	"Other construction workers"	346	309.89	**1.12**	**1.00-1.24**	348.52	0.99	0.89-1.10
35	Bricklayers	115	83.29	**1.38**	**1.14-1.66**	100.64	1.14	0.94-1.37
36	Printers	114	88.21	**1.29**	**1.07-1.55**	120.48	0.95	0.78-1.14
37	Chemical process workers	117	120.86	0.97	0.80-1.16	116.62	1.00	0.83-1.20
38	Food workers	183	162.86	1.12	0.97-1.30	176.81	1.04	0.89-1.20
39	Beverage workers	32	11.48	**2.79**	**1.91-3.93**	34.11	0.94	0.64-1.32
40	Tobacco workers	1	1.87	0.54	0.01-2.98	2.35	0.43	0.01-2.37
41	Glass makers etc	134	133.08	1.01	0.84-1.19	133.42	1.00	0.84-1.19
42	Packers	324	230.01	**1.41**	**1.26-1.57**	316.32	1.02	0.92-1.14
43	Engine operators	197	215.72	0.91	0.79-1.05	223.49	0.88	0.76-1.01
44	Public safety workers	98	138.34	**0.71**	**0.58-0.86**	131.85	**0.74**	**0.60-0.91**
45	Cooks and stewards	76	29.00	**2.62**	**2.06-3.28**	78.17	0.97	0.77-1.22
46	Domestic assistants	3	1.79	1.68	0.35-4.90	2.53	1.18	0.24-3.46
47	Waiters	110	17.99	**6.11**	**5.02-7.37**	95.03	1.16	0.95-1.40
48	Building caretakers	115	107.18	1.07	0.89-1.29	117.98	0.97	0.80-1.17
49	Chimney sweeps	17	7.08	**2.40**	**1.40-3.84**	12.13	1.40	0.82-2.24
50	Hairdressers	40	23.34	**1.71**	**1.22-2.33**	36.30	1.10	0.79-1.50
51	Launderers	16	14.52	1.10	0.63-1.79	13.83	1.16	0.66-1.88
52	Military personnel	69	82.66	0.83	0.65-1.06	79.24	0.87	0.68-1.10
53	"Other workers"	481	357.46	**1.35**	**1.23-1.47**	459.73	1.05	0.95-1.14
54	Economically inactive	909	495.01	**1.84**	**1.72-1.96**	857.58	1.06	0.99-1.13

Table S4. Unadjusted and tobacco and alcohol adjusted SIRs for cancer of the tongue/mouth/pharynx cancer among 7,454,847 women in the Nordic countries, by occupation. Follow up 1961–2005.

			Unadjusted			Adjusted		
No	Occupational category	Obs	Exp	SIR	95% CI	Exp	SIR	95% CI
1	Technical workers, etc	37	51.20	0.72	0.51-1.00	48.74	0.76	0.53-1.05
2	Laboratory assistants	18	26.22	0.69	0.41-1.09	24.04	0.75	0.44-1.18
3	Physicians	12	13.97	0.86	0.44-1.50	13.76	0.87	0.45-1.52
4	Dentists	17	11.77	1.44	0.84-2.31	12.34	1.38	0.80-2.21
5	Nurses	158	196.29	**0.80**	**0.68-0.94**	159.42	0.99	0.84-1.16
6	Assistant nurses	225	246.03	0.91	0.80-1.04	227.97	0.99	0.86-1.12
7	"Other health workers"	106	136.26	**0.78**	**0.64-0.94**	127.38	0.83	0.68-1.01
8	Teachers	329	373.07	**0.88**	**0.79-0.98**	308.10	1.07	0.96-1.19
9	Religious workers etc	105	119.92	0.88	0.72-1.06	106.47	0.99	0.81-1.19
10	Artistic workers	45	25.95	**1.73**	**1.26-2.32**	30.23	**1.49**	**1.09-1.99**
11	Journalists	23	11.09	**2.07**	**1.31-3.11**	11.42	**2.01**	**1.28-3.02**
12	Administrators	79	78.27	1.01	0.80-1.26	84.15	0.94	0.74-1.17
13	Clerical workers	1257	1097.51	**1.15**	**1.08-1.21**	1195.31	1.05	0.99-1.11
14	Sales agents	140	123.78	1.13	0.95-1.33	132.95	1.05	0.89-1.24
15	Shop workers	655	693.60	0.94	0.87-1.02	690.61	0.95	0.88-1.02
16	Farmers	257	322.09	**0.80**	**0.70-0.90**	221.83	**1.16**	**1.02-1.31**
17	Gardeners	283	340.31	**0.83**	**0.74-0.93**	294.33	0.96	0.85-1.08
18	Fishermen	3	2.21	1.36	0.28-3.96	2.24	1.34	0.28-3.91
19	Forestry workers	2	3.71	0.54	0.07-1.95	3.67	0.55	0.07-1.97
22	Transport workers	8	10.64	0.75	0.32-1.48	11.64	0.69	0.30-1.35
23	Drivers	27	20.98	1.29	0.85-1.87	24.48	1.10	0.73-1.60
24	Postal workers	143	155.03	0.92	0.78-1.09	150.77	0.95	0.80-1.12
25	Textile workers	333	340.90	0.98	0.87-1.09	343.60	0.97	0.87-1.08
26	Shoe and leather workers	20	28.86	0.69	0.42-1.07	30.41	0.66	0.40-1.02
27	Smelting workers	9	6.85	1.31	0.60-2.50	7.49	1.20	0.55-2.28
28	Mechanics	70	57.35	1.22	0.95-1.54	66.32	1.06	0.82-1.33
30	Welders	3	2.48	1.21	0.25-3.54	2.67	1.12	0.23-3.28
31	Electrical workers	45	44.77	1.01	0.73-1.35	50.84	0.89	0.65-1.18
32	Wood workers	29	30.59	0.95	0.63-1.36	29.18	0.99	0.67-1.43
33	Painters	2	4.27	0.47	0.06-1.69	4.45	0.45	0.05-1.62
34	"Other construction workers"	7	7.89	0.89	0.36-1.83	10.39	0.67	0.27-1.39
36	Printers	40	33.66	1.19	0.85-1.62	39.88	1.00	0.72-1.37
37	Chemical process workers	24	28.23	0.85	0.54-1.27	28.02	0.86	0.55-1.27
38	Food workers	148	131.98	1.12	0.95-1.32	148.11	1.00	0.84-1.17
39	Beverage workers	10	7.53	1.33	0.64-2.44	10.21	0.98	0.47-1.80
40	Tobacco workers	15	8.43	**1.78**	**1.00-2.93**	12.92	1.16	0.65-1.91
41	Glass makers etc	79	65.53	1.21	0.95-1.50	73.43	1.08	0.85-1.34
42	Packers	125	99.43	**1.26**	**1.05-1.50**	107.97	1.16	0.96-1.38
43	Engine operators	10	8.32	1.20	0.58-2.21	9.45	1.06	0.51-1.95
44	Public safety workers	5	8.47	0.59	0.19-1.38	9.01	0.55	0.18-1.29
45	Cooks and stewards	146	133.57	1.09	0.92-1.29	145.35	1.00	0.85-1.18
46	Domestic assistants	313	361.31	**0.87**	**0.77-0.97**	329.06	0.95	0.85-1.06
47	Waiters	232	138.71	**1.67**	**1.46-1.90**	189.44	**1.22**	**1.07-1.39**
48	Building caretakers	692	626.94	**1.10**	**1.02-1.19**	700.14	0.99	0.92-1.07
50	Hairdressers	69	58.98	1.17	0.91-1.48	65.96	1.05	0.81-1.32
51	Launderers	82	75.64	1.08	0.86-1.35	82.83	0.99	0.79-1.23
53	"Other workers"	285	248.31	**1.15**	**1.02-1.29**	279.71	1.02	0.90-1.14
54	Economically inactive	6442	6543.99	0.98	0.96-1.01	6412.94	1.00	0.98-1.03

Table S5. Unadjusted and tobacco and alcohol adjusted SIRs for cancer of the larynx among 7,447,726 men in the Nordic countries, by occupation. Follow up 1961–2005.

			Unadjusted			Adjusted		
No	Occupational category	Obs	Exp	SIR	95% CI	Exp	SIR	95% CI
1	Technical workers, etc	899	1213.16	**0.74**	**0.69-0.79**	1014.44	**0.89**	**0.83-0.95**
2	Laboratory assistants	11	20.57	**0.53**	**0.27-0.96**	20.33	**0.54**	**0.27-0.97**
3	Physicians	47	78.88	**0.60**	**0.44-0.79**	62.25	0.76	0.55-1.00
4	Dentists	26	30.49	0.85	0.56-1.25	28.13	0.92	0.60-1.35
5	Nurses	0	2.87	0.00	0.00-1.28	2.49	0.00	0.00-1.48
6	Assistant nurses	21	20.01	1.05	0.65-1.60	18.00	1.17	0.72-1.78
7	"Other health workers"	52	61.82	0.84	0.63-1.10	53.12	0.98	0.73-1.28
8	Teachers	253	457.88	**0.55**	**0.49-0.63**	291.14	**0.87**	**0.77-0.98**
9	Religious workers etc	184	258.65	**0.71**	**0.61-0.82**	228.67	**0.80**	**0.69-0.93**
10	Artistic workers	92	83.08	1.11	0.89-1.36	122.64	**0.75**	**0.60-0.92**
11	Journalists	50	39.12	1.28	0.95-1.69	56.95	0.88	0.65-1.16
12	Administrators	847	862.07	0.98	0.92-1.05	938.51	**0.90**	**0.84-0.97**
13	Clerical workers	573	613.77	0.93	0.86-1.01	610.38	0.94	0.86-1.02
14	Sales agents	839	702.38	**1.19**	**1.11-1.28**	842.78	1.00	0.93-1.07
15	Shop workers	579	566.04	1.02	0.94-1.11	643.78	**0.90**	**0.83-0.98**
16	Farmers	1050	2228.40	**0.47**	**0.44-0.50**	1176.21	**0.89**	**0.84-0.95**
17	Gardeners	291	500.21	**0.58**	**0.52-0.65**	324.86	0.90	0.80-1.01
18	Fishermen	241	202.20	**1.19**	**1.05-1.35**	186.62	**1.29**	**1.13-1.47**
19	Forestry workers	255	350.89	**0.73**	**0.64-0.82**	232.76	1.10	0.97-1.24
20	Miners and quarry workers	80	83.17	0.96	0.76-1.20	79.33	1.01	0.80-1.26
21	Seamen	378	206.39	**1.83**	**1.65-2.03**	369.69	1.02	0.92-1.13
22	Transport workers	313	317.08	0.99	0.88-1.10	300.61	1.04	0.93-1.16
23	Drivers	1225	905.25	**1.35**	**1.28-1.43**	1049.05	**1.17**	**1.10-1.24**
24	Postal workers	172	173.00	0.99	0.85-1.15	171.13	1.01	0.86-1.17
25	Textile workers	182	168.34	1.08	0.93-1.25	161.97	1.12	0.97-1.30
26	Shoe and leather workers	87	62.15	**1.40**	**1.12-1.73**	74.62	1.17	0.93-1.44
27	Smelting workers	373	293.37	**1.27**	**1.15-1.41**	304.46	**1.23**	**1.10-1.36**
28	Mechanics	1355	1212.80	**1.12**	**1.06-1.18**	1235.72	**1.10**	**1.04-1.16**
29	Plumbers	149	144.04	1.03	0.87-1.21	161.31	0.92	0.78-1.08
30	Welders	146	128.21	1.14	0.96-1.34	129.17	1.13	0.95-1.33
31	Electrical workers	477	425.23	**1.12**	**1.02-1.23**	425.12	**1.12**	**1.02-1.23**
32	Wood workers	818	996.54	0.82	0.77-0.88	801.96	1.02	0.95-1.09
33	Painters	303	250.61	**1.21**	**1.08-1.35**	298.07	1.02	0.91-1.14
34	"Other construction workers"	750	612.54	**1.22**	**1.14-1.32**	680.19	**1.10**	**1.02-1.18**
35	Bricklayers	167	159.19	1.05	0.90-1.22	188.80	0.88	0.76-1.03
36	Printers	173	143.67	**1.20**	**1.03-1.40**	185.36	0.93	0.80-1.08
37	Chemical process workers	247	214.03	**1.15**	**1.01-1.31**	214.05	**1.15**	**1.01-1.31**
38	Food workers	379	303.68	**1.25**	**1.13-1.38**	326.86	**1.16**	**1.05-1.28**
39	Beverage workers	59	23.52	**2.51**	**1.91-3.24**	54.12	1.09	0.83-1.41
40	Tobacco workers	4	4.05	0.99	0.27-2.53	4.97	0.81	0.22-2.06
41	Glass makers etc	284	234.75	**1.21**	**1.07-1.36**	239.13	**1.19**	**1.05-1.33**
42	Packers	536	408.85	**1.31**	**1.20-1.43**	536.88	1.00	0.92-1.09
43	Engine operators	435	362.80	**1.20**	**1.09-1.32**	377.18	**1.15**	**1.05-1.27**
44	Public safety workers	233	240.06	0.97	0.85-1.10	233.91	1.00	0.87-1.13
45	Cooks and stewards	96	42.73	**2.25**	**1.82-2.74**	96.08	1.00	0.81-1.22
46	Domestic assistants	1	1.50	0.67	0.02-3.71	1.95	0.51	0.01-2.86
47	Waiters	101	30.54	**3.31**	**2.69-4.02**	113.13	0.89	0.73-1.08
48	Building caretakers	254	200.60	**1.27**	**1.12-1.43**	218.20	**1.16**	**1.03-1.32**
49	Chimney sweeps	13	12.43	1.05	0.56-1.79	18.70	0.70	0.37-1.19
50	Hairdressers	66	42.59	**1.55**	**1.20-1.97**	58.51	1.13	0.87-1.44
51	Launderers	25	25.85	0.97	0.63-1.43	24.94	1.00	0.65-1.48
52	Military personnel	130	134.54	0.97	0.81-1.15	131.51	0.99	0.83-1.17
53	"Other workers"	834	662.16	**1.26**	**1.18-1.35**	828.38	1.01	0.94-1.08
54	Economically inactive	1321	957.20	**1.38**	**1.31-1.46**	1446.06	**0.91**	**0.86-0.96**

Table S6. Unadjusted and tobacco and alcohol adjusted SIRs for cancer of the larynx among 7,454,847 women in the Nordic countries, by occupation. Follow up 1961–2005.

			Unadjusted			Adjusted		
No	Occupational category	Obs	Exp	SIR	95% CI	Exp	SIR	95% CI
1	Technical workers, etc	9	9.77	0.92	0.42-1.75	9.66	0.93	0.43-1.77
2	Laboratory assistants	5	5.69	0.88	0.29-2.05	4.64	1.08	0.35-2.52
3	Physicians	1	2.77	0.36	0.01-2.01	2.71	0.37	0.01-2.05
4	Dentists	2	2.39	0.84	0.10-3.03	2.68	0.75	0.09-2.70
5	Nurses	13	42.84	**0.30**	**0.16-0.52**	26.56	**0.49**	**0.26-0.84**
6	Assistant nurses	41	49.18	0.83	0.60-1.13	40.79	1.01	0.72-1.36
7	"Other health workers"	27	28.56	0.95	0.62-1.38	24.01	1.12	0.74-1.64
8	Teachers	34	74.09	**0.46**	**0.32-0.64**	47.12	0.72	0.50-1.01
9	Religious workers etc	10	19.40	**0.52**	**0.25-0.95**	14.70	0.68	0.33-1.25
10	Artistic workers	6	4.99	1.20	0.44-2.62	6.74	0.89	0.33-1.94
11	Journalists	1	1.96	0.51	0.01-2.84	2.10	0.48	0.01-2.65
12	Administrators	23	17.83	1.29	0.82-1.94	21.06	1.09	0.69-1.64
13	Clerical workers	242	242.33	1.00	0.88-1.13	287.49	**0.84**	**0.74-0.95**
14	Sales agents	19	19.08	1.00	0.60-1.55	21.45	0.89	0.53-1.38
15	Shop workers	136	155.03	0.88	0.74-1.04	148.98	0.91	0.77-1.08
16	Farmers	35	78.99	**0.44**	**0.31-0.62**	30.97	1.13	0.79-1.57
17	Gardeners	34	60.07	**0.57**	**0.39-0.79**	33.52	1.01	0.70-1.42
18	Fishermen	1	0.54	1.85	0.05-10.3	0.57	1.77	0.04-9.85
19	Forestry workers	0	0.67	0.00	0.00-5.52	0.64	0.00	0.00-5.77
22	Transport workers	5	1.79	2.79	0.90-6.50	2.24	2.23	0.72-5.21
23	Drivers	9	4.51	2.00	0.91-3.79	6.18	1.46	0.67-2.76
24	Postal workers	21	28.43	0.74	0.46-1.13	26.29	0.80	0.49-1.22
25	Textile workers	52	67.36	0.77	0.58-1.01	68.26	0.76	0.57-1.00
26	Shoe and leather workers	8	5.69	1.41	0.61-2.77	6.20	1.29	0.56-2.54
27	Smelting workers	2	1.42	1.41	0.17-5.10	1.72	1.16	0.14-4.20
28	Mechanics	27	12.14	**2.22**	**1.47-3.24**	16.30	**1.66**	**1.09-2.41**
30	Welders	2	0.41	4.93	0.60-17.8	0.53	3.80	0.46-13.7
31	Electrical workers	21	11.14	**1.89**	**1.17-2.88**	15.15	1.39	0.86-2.12
32	Wood workers	7	6.27	1.12	0.45-2.30	4.96	1.41	0.57-2.91
33	Painters	2	0.74	2.69	0.33-9.71	0.81	2.47	0.30-8.91
34	"Other construction workers"	10	1.36	**7.37**	**3.53-13.6**	5.46	1.83	0.88-3.37
36	Printers	14	6.76	**2.07**	**1.13-3.48**	10.14	1.38	0.75-2.32
37	Chemical process workers	6	6.72	0.89	0.33-1.94	6.14	0.98	0.36-2.13
38	Food workers	46	32.02	**1.44**	**1.05-1.92**	40.04	1.15	0.84-1.53
39	Beverage workers	5	2.17	2.30	0.75-5.37	4.03	1.24	0.40-2.89
40	Tobacco workers	5	2.75	1.82	0.59-4.25	5.96	0.84	0.27-1.96
41	Glass makers etc	18	13.41	1.34	0.80-2.12	16.96	1.06	0.63-1.68
42	Packers	17	17.16	0.99	0.58-1.59	20.83	0.82	0.48-1.31
43	Engine operators	4	1.40	2.86	0.78-7.31	2.56	1.56	0.43-4.00
44	Public safety workers	5	1.39	**3.60**	**1.17-8.40**	1.52	**3.29**	**1.07-7.67**
45	Cooks and stewards	32	20.14	**1.59**	**1.09-2.24**	24.94	1.28	0.88-1.81
46	Domestic assistants	70	70.38	0.99	0.78-1.26	58.26	1.20	0.94-1.52
47	Waiters	55	25.36	**2.17**	**1.63-2.82**	56.03	0.98	0.74-1.28
48	Building caretakers	215	146.14	**1.47**	**1.28-1.68**	186.24	**1.15**	**1.01-1.32**
50	Hairdressers	21	12.15	**1.73**	**1.07-2.64**	17.43	1.20	0.75-1.84
51	Launderers	22	16.70	1.32	0.83-2.00	19.61	1.12	0.70-1.70
53	"Other workers"	81	53.89	**1.50**	**1.19-1.87**	72.47	1.12	0.89-1.39
54	Economically inactive	1254	1288.61	0.97	0.92-1.03	1232.50	1.02	0.96-1.08

Table S7. Unadjusted and tobacco and alcohol adjusted SIRs for cancer of the oesophagus among 7,447,726 men in the Nordic countries, by occupation. Follow up 1961–2005.

			Unadjusted			Adjusted		
No	Occupational category	Obs	Exp	SIR	95% CI	Exp	SIR	95% CI
1	Technical workers, etc	1019	1348.14	**0.76**	**0.71-0.80**	1150.89	**0.89**	**0.83-0.94**
2	Laboratory assistants	20	20.46	0.98	0.60-1.51	19.80	1.01	0.62-1.56
3	Physicians	42	85.03	**0.49**	**0.36-0.67**	68.78	**0.61**	**0.44-0.83**
4	Dentists	20	33.94	**0.59**	**0.36-0.91**	31.41	**0.64**	**0.39-0.98**
5	Nurses	3	3.25	0.92	0.19-2.70	2.80	1.07	0.22-3.13
6	Assistant nurses	20	22.07	0.91	0.55-1.40	20.55	0.97	0.59-1.50
7	"Other health workers"	37	65.42	**0.57**	**0.40-0.78**	57.15	**0.65**	**0.46-0.89**
8	Teachers	272	490.04	**0.56**	**0.49-0.63**	335.62	**0.81**	**0.72-0.91**
9	Religious workers etc	190	289.46	**0.66**	**0.57-0.76**	260.59	**0.73**	**0.63-0.84**
10	Artistic workers	109	90.88	1.20	0.98-1.45	126.64	0.86	0.71-1.04
11	Journalists	50	42.41	1.18	0.87-1.55	58.10	0.86	0.64-1.13
12	Administrators	863	888.10	0.97	0.91-1.04	949.85	**0.91**	**0.85-0.97**
13	Clerical workers	603	662.31	**0.91**	**0.84-0.99**	661.54	**0.91**	**0.84-0.99**
14	Sales agents	809	786.27	1.03	0.96-1.10	921.40	**0.88**	**0.82-0.94**
15	Shop workers	620	559.20	**1.11**	**1.02-1.20**	619.79	1.00	0.92-1.08
16	Farmers	1544	2321.08	**0.67**	**0.63-0.70**	1331.54	**1.16**	**1.10-1.22**
17	Gardeners	417	549.53	**0.76**	**0.69-0.84**	378.13	**1.10**	**1.00-1.21**
18	Fishermen	205	203.89	1.01	0.87-1.15	189.49	1.08	0.94-1.24
19	Forestry workers	352	385.81	0.91	0.82-1.01	266.36	**1.32**	**1.19-1.47**
20	Miners and quarry workers	100	90.63	1.10	0.90-1.34	85.50	1.17	0.95-1.42
21	Seamen	332	201.90	**1.64**	**1.47-1.83**	333.43	1.00	0.89-1.11
22	Transport workers	281	334.77	**0.84**	**0.74-0.94**	319.01	**0.88**	**0.78-0.99**
23	Drivers	1126	933.06	**1.21**	**1.14-1.28**	1049.48	**1.07**	**1.01-1.14**
24	Postal workers	161	184.63	0.87	0.74-1.02	183.72	0.88	0.75-1.02
25	Textile workers	165	182.77	0.90	0.77-1.05	175.97	0.94	0.80-1.09
26	Shoe and leather workers	84	68.38	1.23	0.98-1.52	80.90	1.04	0.83-1.29
27	Smelting workers	326	309.57	1.05	0.94-1.17	313.60	1.04	0.93-1.16
28	Mechanics	1411	1311.32	**1.08**	**1.02-1.13**	1318.21	**1.07**	**1.01-1.13**
29	Plumbers	184	154.59	**1.19**	**1.02-1.38**	168.54	1.09	0.94-1.26
30	Welders	141	144.04	0.98	0.82-1.15	143.82	0.98	0.83-1.16
31	Electrical workers	457	462.25	0.99	0.90-1.08	458.54	1.00	0.91-1.09
32	Wood workers	882	1054.13	0.84	0.78-0.89	864.06	1.02	0.95-1.09
33	Painters	306	268.19	**1.14**	**1.02-1.28**	304.54	1.00	0.90-1.12
34	"Other construction workers"	815	622.68	**1.31**	**1.22-1.40**	676.35	**1.20**	**1.12-1.29**
35	Bricklayers	180	161.83	1.11	0.96-1.29	183.77	0.98	0.84-1.13
36	Printers	200	153.88	**1.30**	**1.13-1.49**	189.94	1.05	0.91-1.21
37	Chemical process workers	223	229.93	0.97	0.85-1.11	226.72	0.98	0.86-1.12
38	Food workers	371	313.22	**1.18**	**1.07-1.31**	334.83	**1.11**	**1.00-1.23**
39	Beverage workers	61	22.47	**2.71**	**2.08-3.49**	43.03	**1.42**	**1.08-1.82**
40	Tobacco workers	5	3.84	1.30	0.42-3.04	4.47	1.12	0.36-2.61
41	Glass makers etc	268	249.45	1.07	0.95-1.21	251.51	1.07	0.94-1.20
42	Packers	616	439.41	**1.40**	**1.29-1.52**	548.56	**1.12**	**1.04-1.22**
43	Engine operators	422	387.66	1.09	0.99-1.20	398.33	1.06	0.96-1.17
44	Public safety workers	219	254.16	**0.86**	**0.75-0.98**	246.99	0.89	0.77-1.01
45	Cooks and stewards	96	45.17	**2.13**	**1.72-2.60**	89.09	1.08	0.87-1.32
46	Domestic assistants	3	1.91	1.57	0.32-4.60	2.39	1.25	0.26-3.67
47	Waiters	100	30.10	**3.32**	**2.70-4.04**	88.40	1.13	0.92-1.38
48	Building caretakers	210	208.05	1.01	0.88-1.16	221.52	0.95	0.82-1.09
49	Chimney sweeps	24	12.94	**1.85**	**1.19-2.76**	18.47	1.30	0.83-1.93
50	Hairdressers	45	45.16	1.00	0.73-1.33	60.61	**0.74**	**0.54-0.99**
51	Launderers	28	28.15	0.99	0.66-1.44	27.40	1.02	0.68-1.48
52	Military personnel	138	142.53	0.97	0.81-1.14	139.77	0.99	0.83-1.17
53	"Other workers"	925	691.30	**1.34**	**1.25-1.43**	815.46	**1.13**	**1.06-1.21**
54	Economically inactive	1474	982.64	**1.50**	**1.42-1.58**	1403.08	**1.05**	**1.00-1.11**

Table S8. Unadjusted and tobacco and alcohol adjusted SIRs for cancer of the oesophagus among 7,454,847 women in the Nordic countries, by occupation. Follow up 1961–2005.

			Unadjusted			Adjusted		
No	Occupational category	Obs	Exp	SIR	95% CI	Exp	SIR	95% CI
1	Technical workers, etc	23	26.79	0.86	0.54-1.29	24.38	0.94	0.60-1.42
2	Laboratory assistants	10	12.84	0.78	0.37-1.43	11.66	0.86	0.41-1.58
3	Physicians	8	6.84	1.17	0.51-2.31	6.59	1.21	0.52-2.39
4	Dentists	6	7.60	0.79	0.29-1.72	7.63	0.79	0.29-1.71
5	Nurses	73	107.34	**0.68**	**0.53-0.86**	89.17	0.82	0.64-1.03
6	Assistant nurses	103	129.04	**0.80**	**0.65-0.97**	118.33	0.87	0.71-1.06
7	"Other health workers"	60	74.10	0.81	0.62-1.04	69.66	0.86	0.66-1.11
8	Teachers	162	204.19	**0.79**	**0.68-0.93**	169.76	0.95	0.81-1.11
9	Religious workers etc	49	56.00	0.88	0.65-1.16	48.24	1.02	0.75-1.34
10	Artistic workers	17	14.10	1.21	0.70-1.93	16.05	1.06	0.62-1.70
11	Journalists	6	6.10	0.98	0.36-2.14	6.06	0.99	0.36-2.16
12	Administrators	50	44.52	1.12	0.83-1.48	46.24	1.08	0.80-1.43
13	Clerical workers	654	617.86	1.06	0.98-1.14	658.46	0.99	0.92-1.07
14	Sales agents	81	80.05	1.01	0.80-1.26	84.83	0.95	0.76-1.19
15	Shop workers	426	432.08	0.99	0.89-1.08	430.20	0.99	0.90-1.09
16	Farmers	206	239.03	**0.86**	**0.75-0.99**	185.76	1.11	0.96-1.27
17	Gardeners	286	304.27	0.94	0.83-1.06	263.92	1.08	0.96-1.22
18	Fishermen	1	1.40	0.71	0.02-3.97	1.38	0.73	0.02-4.05
19	Forestry workers	2	2.32	0.86	0.10-3.11	2.26	0.88	0.11-3.20
22	Transport workers	5	8.11	0.62	0.20-1.44	8.22	0.61	0.20-1.42
23	Drivers	16	11.06	1.45	0.83-2.35	12.52	1.28	0.73-2.08
24	Postal workers	95	95.78	0.99	0.80-1.21	91.26	1.04	0.84-1.27
25	Textile workers	240	236.84	1.01	0.89-1.15	233.58	1.03	0.90-1.17
26	Shoe and leather workers	24	20.78	1.16	0.74-1.72	21.25	1.13	0.72-1.68
27	Smelting workers	7	4.48	1.56	0.63-3.22	4.64	1.51	0.61-3.11
28	Mechanics	32	33.67	0.95	0.65-1.34	38.01	0.84	0.58-1.19
30	Welders	0	1.21	0.00	0.00-3.04	1.24	0.00	0.00-2.98
31	Electrical workers	26	24.85	1.05	0.68-1.53	26.53	0.98	0.64-1.44
32	Wood workers	18	25.44	0.71	0.42-1.12	23.47	0.77	0.45-1.21
33	Painters	5	3.12	1.60	0.52-3.74	3.05	1.64	0.53-3.82
34	"Other construction workers"	10	8.81	1.14	0.54-2.09	9.08	1.10	0.53-2.03
36	Printers	23	21.89	1.05	0.67-1.58	24.72	0.93	0.59-1.40
37	Chemical process workers	22	18.33	1.20	0.75-1.82	18.68	1.18	0.74-1.78
38	Food workers	101	89.81	1.12	0.92-1.37	96.02	1.05	0.86-1.28
39	Beverage workers	7	5.39	1.30	0.52-2.68	6.03	1.16	0.47-2.39
40	Tobacco workers	7	5.49	1.28	0.51-2.63	6.70	1.04	0.42-2.15
41	Glass makers etc	51	43.62	1.17	0.87-1.54	47.24	1.08	0.80-1.42
42	Packers	80	67.64	1.18	0.94-1.47	70.79	1.13	0.90-1.41
43	Engine operators	7	5.92	1.18	0.48-2.44	5.92	1.18	0.48-2.44
44	Public safety workers	3	4.41	0.68	0.14-1.99	4.56	0.66	0.14-1.92
45	Cooks and stewards	96	93.19	1.03	0.83-1.26	99.34	0.97	0.78-1.18
46	Domestic assistants	197	210.87	0.93	0.81-1.07	190.17	1.04	0.90-1.19
47	Waiters	119	87.96	**1.35**	**1.12-1.62**	114.65	1.04	0.86-1.24
48	Building caretakers	441	418.80	1.05	0.96-1.16	448.29	0.98	0.89-1.08
50	Hairdressers	38	35.59	1.07	0.76-1.47	38.50	0.99	0.70-1.35
51	Launderers	67	51.81	**1.29**	**1.00-1.64**	56.64	1.18	0.92-1.50
53	"Other workers"	155	152.66	1.02	0.86-1.19	163.43	0.95	0.80-1.11
54	Economically inactive	4599	4563.58	1.01	0.98-1.04	4412.71	**1.04**	**1.01-1.07**

Table S9. Unadjusted and tobacco and alcohol adjusted SIRs for liver cancer among 7,447,726 men in the Nordic countries, by occupation. Follow up 1961–2005.

			Unadjusted			Adjusted		
No	Occupational category	Obs	Exp	SIR	95% CI	Exp	SIR	95% CI
1	Technical workers, etc	1056	1206.95	**0.87**	**0.82-0.93**	1033.44	1.02	0.96-1.09
2	Laboratory assistants	16	15.26	1.05	0.60-1.70	15.19	1.05	0.60-1.71
3	Physicians	64	70.83	0.90	0.70-1.15	55.41	1.15	0.89-1.47
4	Dentists	28	28.47	0.98	0.65-1.42	27.03	1.04	0.69-1.50
5	Nurses	2	2.42	0.83	0.10-2.99	2.08	0.96	0.12-3.47
6	Assistant nurses	18	18.45	0.98	0.58-1.54	17.35	1.04	0.61-1.64
7	"Other health workers"	59	58.62	1.01	0.77-1.30	50.29	1.17	0.89-1.51
8	Teachers	283	409.60	**0.69**	**0.61-0.78**	272.71	1.04	0.92-1.17
9	Religious workers etc	243	252.66	0.96	0.84-1.09	232.06	1.05	0.92-1.19
10	Artistic workers	114	80.87	**1.41**	**1.16-1.69**	121.87	0.94	0.77-1.12
11	Journalists	74	37.94	**1.95**	**1.53-2.45**	57.28	**1.29**	**1.01-1.62**
12	Administrators	874	760.63	**1.15**	**1.07-1.23**	844.45	1.03	0.97-1.11
13	Clerical workers	593	583.14	1.02	0.94-1.10	600.51	0.99	0.91-1.07
14	Sales agents	987	720.30	**1.37**	**1.29-1.46**	884.79	**1.12**	**1.05-1.19**
15	Shop workers	530	459.39	**1.15**	**1.06-1.26**	534.56	0.99	0.91-1.08
16	Farmers	1065	2243.79	**0.47**	**0.45-0.50**	1238.03	**0.86**	**0.81-0.91**
17	Gardeners	345	524.83	**0.66**	**0.59-0.73**	348.71	0.99	0.89-1.10
18	Fishermen	111	143.19	**0.78**	**0.64-0.93**	131.27	0.85	0.70-1.02
19	Forestry workers	265	382.96	**0.69**	**0.61-0.78**	263.63	1.01	0.89-1.13
20	Miners and quarry workers	100	85.33	1.17	0.95-1.43	80.52	**1.24**	**1.01-1.51**
21	Seamen	270	150.02	**1.80**	**1.59-2.03**	272.45	0.99	0.88-1.12
22	Transport workers	312	305.52	1.02	0.91-1.14	293.83	1.06	0.95-1.19
23	Drivers	1006	824.67	**1.22**	**1.15-1.30**	959.21	1.05	0.98-1.12
24	Postal workers	168	164.29	1.02	0.87-1.19	167.28	1.00	0.86-1.17
25	Textile workers	166	170.26	0.97	0.83-1.14	167.65	0.99	0.85-1.15
26	Shoe and leather workers	72	66.43	1.08	0.85-1.36	83.87	0.86	0.67-1.08
27	Smelting workers	277	278.19	1.00	0.88-1.12	284.43	0.97	0.86-1.10
28	Mechanics	1172	1172.00	1.00	0.94-1.06	1194.98	0.98	0.93-1.04
29	Plumbers	192	140.26	**1.37**	**1.18-1.58**	157.30	**1.22**	**1.05-1.41**
30	Welders	120	128.73	0.93	0.77-1.11	131.88	0.91	0.75-1.09
31	Electrical workers	412	402.97	1.02	0.93-1.13	408.47	1.01	0.91-1.11
32	Wood workers	797	981.23	**0.81**	**0.76-0.87**	797.20	1.00	0.93-1.07
33	Painters	257	248.72	1.03	0.91-1.17	291.61	0.88	0.78-1.00
34	"Other construction workers"	589	591.59	1.00	0.92-1.08	665.15	**0.89**	**0.82-0.96**
35	Bricklayers	166	144.70	1.15	0.98-1.34	169.76	0.98	0.83-1.14
36	Printers	184	132.66	**1.39**	**1.19-1.60**	173.98	1.06	0.91-1.22
37	Chemical process workers	195	210.64	0.93	0.80-1.07	208.58	0.93	0.81-1.08
38	Food workers	260	263.97	0.98	0.87-1.11	287.50	0.90	0.80-1.02
39	Beverage workers	49	19.60	**2.50**	**1.85-3.30**	48.04	1.02	0.75-1.35
40	Tobacco workers	6	3.06	1.96	0.72-4.27	3.85	1.56	0.57-3.39
41	Glass makers etc	214	229.18	0.93	0.81-1.07	232.57	0.92	0.80-1.05
42	Packers	511	411.35	**1.24**	**1.14-1.36**	532.06	0.96	0.88-1.05
43	Engine operators	403	356.64	**1.13**	**1.02-1.25**	376.83	1.07	0.97-1.18
44	Public safety workers	256	228.76	1.12	0.99-1.27	223.53	**1.15**	**1.01-1.29**
45	Cooks and stewards	91	35.03	**2.60**	**2.09-3.19**	79.62	1.14	0.92-1.40
46	Domestic assistants	4	1.77	2.26	0.62-5.78	2.35	1.70	0.46-4.36
47	Waiters	101	24.00	**4.21**	**3.43-5.12**	94.77	1.07	0.87-1.30
48	Building caretakers	209	199.15	1.05	0.91-1.20	217.31	0.96	0.84-1.10
49	Chimney sweeps	19	12.11	1.57	0.94-2.45	18.70	1.02	0.61-1.59
50	Hairdressers	57	39.98	**1.43**	**1.08-1.85**	58.11	0.98	0.74-1.27
51	Launderers	29	25.62	1.13	0.76-1.63	24.61	1.18	0.79-1.69
52	Military personnel	105	117.19	0.90	0.73-1.08	117.37	0.89	0.73-1.08
53	"Other workers"	743	595.18	**1.25**	**1.16-1.34**	746.00	1.00	0.93-1.07
54	Economically inactive	1489	966.90	**1.54**	**1.46-1.62**	1482.45	1.00	0.95-1.06

Table S10. Unadjusted and tobacco and alcohol adjusted SIRs for liver cancer among 7,454,847 women in the Nordic countries, by occupation. Follow up 1961–2005.

			Unadjusted			Adjusted		
No	Occupational category	Obs	Exp	SIR	95% CI	Exp	SIR	95% CI
1	Technical workers, etc	28	36.30	0.77	0.51-1.11	33.72	0.83	0.55-1.20
2	Laboratory assistants	15	15.62	0.96	0.54-1.58	15.09	0.99	0.56-1.64
3	Physicians	6	8.99	0.67	0.24-1.45	8.82	0.68	0.25-1.48
4	Dentists	9	9.32	0.97	0.44-1.83	9.85	0.91	0.42-1.73
5	Nurses	126	137.30	0.92	0.76-1.09	117.88	1.07	0.89-1.27
6	Assistant nurses	163	172.81	0.94	0.80-1.10	162.54	1.00	0.85-1.17
7	"Other health workers"	91	92.16	0.99	0.79-1.21	87.31	1.04	0.84-1.28
8	Teachers	196	263.96	**0.74**	**0.64-0.85**	229.15	**0.86**	**0.74-0.98**
9	Religious workers etc	59	76.97	**0.77**	**0.58-0.99**	70.20	0.84	0.64-1.08
10	Artistic workers	22	18.19	1.21	0.76-1.83	20.31	1.08	0.68-1.64
11	Journalists	5	8.04	0.62	0.20-1.45	8.08	0.62	0.20-1.44
12	Administrators	44	55.02	0.80	0.58-1.07	56.52	0.78	0.57-1.05
13	Clerical workers	734	777.89	0.94	0.88-1.01	838.31	**0.88**	**0.81-0.94**
14	Sales agents	94	102.58	0.92	0.74-1.12	107.77	0.87	0.70-1.07
15	Shop workers	554	541.60	1.02	0.94-1.11	542.30	1.02	0.94-1.11
16	Farmers	167	252.43	**0.66**	**0.56-0.77**	202.72	**0.82**	**0.70-0.96**
17	Gardeners	284	343.67	**0.83**	**0.73-0.93**	295.42	0.96	0.85-1.08
18	Fishermen	2	1.52	1.31	0.16-4.74	1.55	1.29	0.16-4.65
19	Forestry workers	2	2.88	0.70	0.08-2.51	2.84	0.70	0.09-2.54
22	Transport workers	13	10.08	1.29	0.69-2.21	10.99	1.18	0.63-2.02
23	Drivers	15	14.48	1.04	0.58-1.71	15.99	0.94	0.53-1.55
24	Postal workers	135	122.56	1.10	0.92-1.30	120.81	1.12	0.94-1.32
25	Textile workers	314	300.52	1.04	0.93-1.17	301.08	1.04	0.93-1.17
26	Shoe and leather workers	31	26.11	1.19	0.81-1.69	27.63	1.12	0.76-1.59
27	Smelting workers	12	5.69	**2.11**	**1.09-3.68**	6.15	**1.95**	**1.01-3.41**
28	Mechanics	51	44.80	1.14	0.85-1.50	50.76	1.00	0.75-1.32
30	Welders	4	1.63	2.46	0.67-6.30	1.79	2.24	0.61-5.74
31	Electrical workers	35	31.80	1.10	0.77-1.53	34.39	1.02	0.71-1.42
32	Wood workers	21	29.28	0.72	0.44-1.10	27.93	0.75	0.47-1.15
33	Painters	5	3.84	1.30	0.42-3.04	3.99	1.25	0.41-2.92
34	"Other construction workers"	13	9.40	1.38	0.74-2.36	13.38	0.97	0.52-1.66
36	Printers	38	27.49	1.38	0.98-1.90	32.16	1.18	0.84-1.62
37	Chemical process workers	23	22.88	1.01	0.64-1.51	23.31	0.99	0.63-1.48
38	Food workers	105	104.64	1.00	0.82-1.21	111.05	0.95	0.77-1.14
39	Beverage workers	9	6.59	1.37	0.62-2.59	7.24	1.24	0.57-2.36
40	Tobacco workers	13	6.41	**2.03**	**1.08-3.47**	7.65	1.70	0.90-2.90
41	Glass makers etc	69	55.01	1.25	0.98-1.59	61.12	1.13	0.88-1.43
42	Packers	92	85.14	1.08	0.87-1.33	91.57	1.00	0.81-1.23
43	Engine operators	10	7.18	1.39	0.67-2.56	8.56	1.17	0.56-2.15
44	Public safety workers	2	5.44	0.37	0.04-1.33	5.76	0.35	0.04-1.25
45	Cooks and stewards	126	117.75	1.07	0.89-1.27	126.66	0.99	0.83-1.18
46	Domestic assistants	225	270.57	**0.83**	**0.73-0.95**	254.12	0.89	0.77-1.01
47	Waiters	155	114.28	**1.36**	**1.15-1.59**	144.80	1.07	0.91-1.25
48	Building caretakers	600	495.38	**1.21**	**1.12-1.31**	546.34	**1.10**	**1.01-1.19**
50	Hairdressers	52	45.35	1.15	0.86-1.50	51.91	1.00	0.75-1.31
51	Launderers	84	66.06	**1.27**	**1.01-1.57**	72.59	1.16	0.92-1.43
53	"Other workers"	179	182.53	0.98	0.84-1.14	196.65	0.91	0.78-1.05
54	Economically inactive	5942	5843.19	1.02	0.99-1.04	5801.59	**1.02**	**1.00-1.05**

Table S11. Unadjusted and tobacco and alcohol adjusted SIRs for colon cancer among 7,447,726 men in the Nordic countries, by occupation. Follow up 1961–2005.

			Unadjusted			Adjusted		
No	Occupational category	Obs	Exp	SIR	95% CI	Exp	SIR	95% CI
1	Technical workers, etc	7855	7196.47	**1.09**	**1.07-1.12**	7085.85	**1.11**	**1.08-1.13**
2	Laboratory assistants	111	109.76	1.01	0.83-1.22	111.88	0.99	0.82-1.19
3	Physicians	543	470.36	**1.15**	**1.06-1.26**	457.77	**1.19**	**1.09-1.29**
4	Dentists	217	192.98	1.12	0.98-1.28	193.87	1.12	0.98-1.28
5	Nurses	18	15.79	1.14	0.68-1.80	15.64	1.15	0.68-1.82
6	Assistant nurses	114	119.26	0.96	0.79-1.15	119.45	0.95	0.79-1.15
7	"Other health workers"	338	346.44	0.98	0.87-1.09	340.98	0.99	0.89-1.10
8	Teachers	2694	2649.71	1.02	0.98-1.06	2485.64	**1.08**	**1.04-1.13**
9	Religious workers etc	1875	1578.92	**1.19**	**1.13-1.24**	1574.49	**1.19**	**1.14-1.25**
10	Artistic workers	554	496.88	**1.11**	**1.02-1.21**	548.17	1.01	0.93-1.10
11	Journalists	266	228.04	**1.17**	**1.03-1.32**	251.05	1.06	0.94-1.20
12	Administrators	5971	4956.34	**1.20**	**1.17-1.24**	5163.65	**1.16**	**1.13-1.19**
13	Clerical workers	4194	3726.21	**1.13**	**1.09-1.16**	3810.66	**1.10**	**1.07-1.14**
14	Sales agents	5036	4431.49	**1.14**	**1.10-1.17**	4701.65	**1.07**	**1.04-1.10**
15	Shop workers	3279	2994.53	**1.09**	**1.06-1.13**	3147.68	**1.04**	**1.01-1.08**
16	Farmers	10023	13008.81	**0.77**	**0.75-0.79**	11632.7	**0.86**	**0.84-0.88**
17	Gardeners	2607	3186.54	**0.82**	**0.79-0.85**	2988.46	**0.87**	**0.84-0.91**
18	Fishermen	1271	1358.75	**0.94**	**0.88-0.99**	1370.08	**0.93**	**0.88-0.98**
19	Forestry workers	1643	2195.76	**0.75**	**0.71-0.79**	2063.04	**0.80**	**0.76-0.84**
20	Miners and quarry workers	494	523.56	0.94	0.86-1.03	526.08	0.94	0.86-1.03
21	Seamen	1397	1267.08	**1.10**	**1.05-1.16**	1430.78	0.98	0.93-1.03
22	Transport workers	1961	1876.29	**1.05**	**1.00-1.09**	1899.74	1.03	0.99-1.08
23	Drivers	5429	5015.29	**1.08**	**1.05-1.11**	5277.59	**1.03**	**1.00-1.06**
24	Postal workers	1078	997.80	**1.08**	**1.02-1.15**	1016.26	**1.06**	**1.00-1.13**
25	Textile workers	1128	1057.88	**1.07**	**1.00-1.13**	1071.41	1.05	0.99-1.12
26	Shoe and leather workers	457	399.99	**1.14**	**1.04-1.25**	424.84	1.08	0.98-1.18
27	Smelting workers	1728	1709.05	1.01	0.96-1.06	1754.62	0.98	0.94-1.03
28	Mechanics	7385	7236.11	**1.02**	**1.00-1.04**	7407.65	1.00	0.97-1.02
29	Plumbers	912	836.60	**1.09**	**1.02-1.16**	874.04	1.04	0.98-1.11
30	Welders	745	794.40	0.94	0.87-1.01	810.21	**0.92**	**0.85-0.99**
31	Electrical workers	2615	2549.50	1.03	0.99-1.07	2604.22	1.00	0.97-1.04
32	Wood workers	5478	6067.36	**0.90**	**0.88-0.93**	5930.05	**0.92**	**0.90-0.95**
33	Painters	1510	1484.62	1.02	0.97-1.07	1559.39	0.97	0.92-1.02
34	"Other construction workers"	3119	3307.44	**0.94**	**0.91-0.98**	3440.19	**0.91**	**0.87-0.94**
35	Bricklayers	848	898.27	0.94	0.88-1.01	947.84	**0.89**	**0.84-0.96**
36	Printers	958	834.87	**1.15**	**1.08-1.22**	898.94	**1.07**	**1.00-1.14**
37	Chemical process workers	1268	1332.33	0.95	0.90-1.01	1357.21	**0.93**	**0.88-0.99**
38	Food workers	1672	1770.99	**0.94**	**0.90-0.99**	1835.12	**0.91**	**0.87-0.96**
39	Beverage workers	142	117.17	**1.21**	**1.02-1.43**	144.49	0.98	0.83-1.16
40	Tobacco workers	29	22.29	1.30	0.87-1.87	23.79	1.22	0.82-1.75
41	Glass makers etc	1318	1369.94	0.96	0.91-1.02	1400.95	**0.94**	**0.89-0.99**
42	Packers	2688	2509.54	**1.07**	**1.03-1.11**	2691.91	1.00	0.96-1.04
43	Engine operators	2126	2058.03	1.03	0.99-1.08	2120.98	1.00	0.96-1.05
44	Public safety workers	1635	1376.07	**1.19**	**1.13-1.25**	1401.61	**1.17**	**1.11-1.22**
45	Cooks and stewards	259	269.57	0.96	0.85-1.09	317.37	**0.82**	**0.72-0.92**
46	Domestic assistants	11	9.79	1.12	0.56-2.01	10.55	1.04	0.52-1.87
47	Waiters	220	167.96	**1.31**	**1.14-1.50**	221.59	0.99	0.87-1.13
48	Building caretakers	1188	1106.59	**1.07**	**1.01-1.14**	1151.39	1.03	0.97-1.09
49	Chimney sweeps	104	68.82	**1.51**	**1.23-1.83**	76.47	**1.36**	**1.11-1.65**
50	Hairdressers	274	258.89	1.06	0.94-1.19	281.55	0.97	0.86-1.10
51	Launderers	167	161.24	1.04	0.88-1.21	162.39	1.03	0.88-1.20
52	Military personnel	902	805.87	**1.12**	**1.05-1.20**	822.18	**1.10**	**1.03-1.17**
53	"Other workers"	3818	4010.79	**0.95**	**0.92-0.98**	4262.55	**0.90**	**0.87-0.93**
54	Economically inactive	4765	4902.00	0.97	0.94-1.00	5440.21	**0.88**	**0.85-0.90**

Table S12. Unadjusted and tobacco and alcohol adjusted SIRs for colon cancer among 7,454,847 women in the Nordic countries, by occupation. Follow up 1961–2005.

			Unadjusted			Adjusted		
No	Occupational category	Obs	Exp	SIR	95% CI	Exp	SIR	95% CI
1	Technical workers, etc	401	376.68	1.06	0.96-1.17	372.63	1.08	0.97-1.19
2	Laboratory assistants	163	179.60	0.91	0.77-1.06	177.69	0.92	0.78-1.07
3	Physicians	109	101.60	1.07	0.88-1.29	101.60	1.07	0.88-1.29
4	Dentists	104	96.34	1.08	0.88-1.31	97.70	1.06	0.87-1.29
5	Nurses	1613	1619.64	1.00	0.95-1.05	1575.73	1.02	0.97-1.08
6	Assistant nurses	1908	1934.38	0.99	0.94-1.03	1911.35	1.00	0.95-1.04
7	"Other health workers"	1123	1127.44	1.00	0.94-1.06	1128.72	0.99	0.94-1.06
8	Teachers	3020	2982.81	1.01	0.98-1.05	2913.86	**1.04**	**1.00-1.07**
9	Religious workers etc	837	835.73	1.00	0.93-1.07	823.00	1.02	0.95-1.09
10	Artistic workers	195	210.99	0.92	0.80-1.06	215.53	0.90	0.78-1.04
11	Journalists	74	81.58	0.91	0.71-1.14	82.33	0.90	0.71-1.13
12	Administrators	750	674.24	**1.11**	**1.03-1.20**	679.91	**1.10**	**1.03-1.19**
13	Clerical workers	9885	9127.16	**1.08**	**1.06-1.11**	9310.89	**1.06**	**1.04-1.08**
14	Sales agents	1155	1096.16	1.05	0.99-1.12	1113.37	1.04	0.98-1.10
15	Shop workers	6869	6588.79	**1.04**	**1.02-1.07**	6634.22	**1.04**	**1.01-1.06**
16	Farmers	2838	3230.49	**0.88**	**0.85-0.91**	3059.64	**0.93**	**0.89-0.96**
17	Gardeners	2664	3036.36	**0.88**	**0.84-0.91**	2939.24	**0.91**	**0.87-0.94**
18	Fishermen	19	20.92	0.91	0.55-1.42	20.93	0.91	0.55-1.42
19	Forestry workers	32	35.47	0.90	0.62-1.27	35.73	0.90	0.61-1.26
22	Transport workers	95	83.98	1.13	0.92-1.38	85.32	1.11	0.90-1.36
23	Drivers	160	170.18	0.94	0.80-1.10	173.18	0.92	0.79-1.08
24	Postal workers	1484	1396.87	**1.06**	**1.01-1.12**	1396.10	**1.06**	**1.01-1.12**
25	Textile workers	3474	3230.74	**1.08**	**1.04-1.11**	3264.68	**1.06**	**1.03-1.10**
26	Shoe and leather workers	246	263.29	0.93	0.82-1.06	266.05	0.92	0.81-1.05
27	Smelting workers	45	54.02	0.83	0.61-1.11	54.90	0.82	0.60-1.10
28	Mechanics	476	473.71	1.00	0.92-1.10	485.03	0.98	0.89-1.07
30	Welders	14	16.98	0.82	0.45-1.38	17.28	0.81	0.44-1.36
31	Electrical workers	368	372.31	0.99	0.89-1.09	379.57	0.97	0.87-1.07
32	Wood workers	206	235.23	0.88	0.76-1.00	234.22	0.88	0.76-1.01
33	Painters	38	33.48	1.14	0.80-1.56	33.91	1.12	0.79-1.54
34	"Other construction workers"	58	58.95	0.98	0.75-1.27	62.93	0.92	0.70-1.19
36	Printers	327	286.26	**1.14**	**1.02-1.27**	295.91	1.11	0.99-1.23
37	Chemical process workers	292	257.86	**1.13**	**1.01-1.27**	261.21	1.12	0.99-1.25
38	Food workers	1218	1241.88	0.98	0.93-1.04	1256.04	0.97	0.92-1.03
39	Beverage workers	66	68.16	0.97	0.75-1.23	69.62	0.95	0.73-1.21
40	Tobacco workers	71	78.25	0.91	0.71-1.14	81.08	0.88	0.68-1.10
41	Glass makers etc	620	572.68	**1.08**	**1.00-1.17**	585.42	1.06	0.98-1.15
42	Packers	939	906.38	1.04	0.97-1.10	923.09	1.02	0.95-1.08
43	Engine operators	46	56.49	0.81	0.60-1.09	58.14	0.79	0.58-1.06
44	Public safety workers	79	73.76	1.07	0.85-1.33	73.94	1.07	0.85-1.33
45	Cooks and stewards	1260	1259.80	1.00	0.95-1.06	1294.25	0.97	0.92-1.03
46	Domestic assistants	3129	3283.35	**0.95**	**0.92-0.99**	3246.46	0.96	0.93-1.00
47	Waiters	1408	1318.99	**1.07**	**1.01-1.13**	1391.28	1.01	0.96-1.07
48	Building caretakers	5579	5893.91	**0.95**	**0.92-0.97**	6033.75	**0.92**	**0.90-0.95**
50	Hairdressers	550	506.58	**1.09**	**1.00-1.18**	519.97	1.06	0.97-1.15
51	Launderers	723	747.01	0.97	0.90-1.04	762.55	0.95	0.88-1.02
53	"Other workers"	2356	2359.55	1.00	0.96-1.04	2407.58	0.98	0.94-1.02
54	Economically inactive	63182	63603.29	0.99	0.98-1.00	63793.40	0.99	0.98-1.00

Table S13. Unadjusted and tobacco and alcohol adjusted SIRs for rectal cancer among 7,447,726 men in the Nordic countries, by occupation. Follow up 1961–2005.

			Unadjusted			Adjusted		
No	Occupational category	Obs	Exp	SIR	95% CI	Exp	SIR	95% CI
1	Technical workers, etc	5039	5018.46	1.00	0.98-1.03	4927.56	1.02	0.99-1.05
2	Laboratory assistants	84	75.12	1.12	0.89-1.38	76.01	1.11	0.88-1.37
3	Physicians	278	320.58	**0.87**	**0.77-0.98**	312.22	0.89	0.79-1.00
4	Dentists	134	130.24	1.03	0.86-1.22	129.54	1.03	0.87-1.23
5	Nurses	9	11.66	0.77	0.35-1.47	11.53	0.78	0.36-1.48
6	Assistant nurses	70	82.00	0.85	0.67-1.08	81.51	0.86	0.67-1.09
7	"Other health workers"	239	241.98	0.99	0.87-1.12	238.61	1.00	0.88-1.14
8	Teachers	1605	1823.90	**0.88**	**0.84-0.92**	1711.24	**0.94**	**0.89-0.99**
9	Religious workers etc	1098	1091.84	1.01	0.95-1.07	1081.37	1.02	0.96-1.08
10	Artistic workers	361	344.15	1.05	0.94-1.16	371.01	0.97	0.88-1.08
11	Journalists	149	158.57	0.94	0.79-1.10	170.01	0.88	0.74-1.03
12	Administrators	3611	3363.60	**1.07**	**1.04-1.11**	3459.56	**1.04**	**1.01-1.08**
13	Clerical workers	2602	2532.75	1.03	0.99-1.07	2562.38	1.02	0.98-1.06
14	Sales agents	3385	3039.31	**1.11**	**1.08-1.15**	3171.36	**1.07**	**1.03-1.10**
15	Shop workers	2151	2072.42	1.04	0.99-1.08	2144.45	1.00	0.96-1.05
16	Farmers	7632	8912.84	**0.86**	**0.84-0.88**	8081.53	**0.94**	**0.92-0.97**
17	Gardeners	2034	2159.35	**0.94**	**0.90-0.98**	2040.23	1.00	0.95-1.04
18	Fishermen	791	851.73	0.93	0.86-1.00	853.86	**0.93**	**0.86-0.99**
19	Forestry workers	1312	1506.14	**0.87**	**0.82-0.92**	1426.44	**0.92**	**0.87-0.97**
20	Miners and quarry workers	306	357.81	**0.86**	**0.76-0.96**	359.31	**0.85**	**0.76-0.95**
21	Seamen	885	825.34	**1.07**	**1.00-1.15**	928.94	0.95	0.89-1.02
22	Transport workers	1331	1279.02	1.04	0.99-1.10	1286.07	1.03	0.98-1.09
23	Drivers	3753	3496.38	**1.07**	**1.04-1.11**	3634.94	**1.03**	**1.00-1.07**
24	Postal workers	687	687.83	1.00	0.93-1.08	695.51	0.99	0.92-1.06
25	Textile workers	783	715.52	**1.09**	**1.02-1.17**	718.70	**1.09**	**1.01-1.17**
26	Shoe and leather workers	299	270.08	1.11	0.98-1.24	281.46	1.06	0.95-1.19
27	Smelting workers	1185	1172.94	1.01	0.95-1.07	1193.96	0.99	0.94-1.05
28	Mechanics	4996	5000.73	1.00	0.97-1.03	5080.64	0.98	0.96-1.01
29	Plumbers	653	583.68	**1.12**	**1.03-1.21**	602.75	**1.08**	**1.00-1.17**
30	Welders	596	554.40	1.08	0.99-1.17	561.74	1.06	0.98-1.15
31	Electrical workers	1789	1758.72	1.02	0.97-1.07	1782.00	1.00	0.96-1.05
32	Wood workers	3988	4118.07	0.97	0.94-1.00	4025.52	0.99	0.96-1.02
33	Painters	1130	1023.39	**1.10**	**1.04-1.17**	1064.03	**1.06**	**1.00-1.13**
34	"Other construction workers"	2303	2329.63	0.99	0.95-1.03	2396.50	0.96	0.92-1.00
35	Bricklayers	698	617.39	**1.13**	**1.05-1.22**	643.63	**1.08**	**1.01-1.17**
36	Printers	641	577.99	**1.11**	**1.02-1.20**	610.67	1.05	0.97-1.13
37	Chemical process workers	968	903.81	**1.07**	**1.00-1.14**	916.16	1.06	0.99-1.13
38	Food workers	1259	1196.92	1.05	0.99-1.11	1228.81	1.02	0.97-1.08
39	Beverage workers	111	83.10	**1.34**	**1.10-1.61**	97.27	1.14	0.94-1.37
40	Tobacco workers	12	15.26	0.79	0.41-1.37	16.03	0.75	0.39-1.31
41	Glass makers etc	981	949.68	1.03	0.97-1.10	964.44	1.02	0.95-1.08
42	Packers	1918	1714.83	**1.12**	**1.07-1.17**	1816.87	**1.06**	**1.01-1.10**
43	Engine operators	1509	1444.85	1.04	0.99-1.10	1474.30	1.02	0.97-1.08
44	Public safety workers	1055	954.12	**1.11**	**1.04-1.17**	964.77	**1.09**	**1.03-1.16**
45	Cooks and stewards	205	179.05	1.14	0.99-1.31	207.51	0.99	0.86-1.13
46	Domestic assistants	8	7.13	1.12	0.48-2.21	7.49	1.07	0.46-2.10
47	Waiters	159	115.92	**1.37**	**1.17-1.60**	146.91	1.08	0.92-1.26
48	Building caretakers	786	778.66	1.01	0.94-1.08	800.77	0.98	0.91-1.05
49	Chimney sweeps	51	48.68	1.05	0.78-1.38	52.73	0.97	0.72-1.27
50	Hairdressers	200	176.19	1.14	0.98-1.30	187.13	1.07	0.93-1.23
51	Launderers	98	109.79	0.89	0.72-1.09	110.20	0.89	0.72-1.08
52	Military personnel	568	544.64	1.04	0.96-1.13	550.09	1.03	0.95-1.12
53	"Other workers"	2778	2692.21	1.03	0.99-1.07	2824.19	0.98	0.95-1.02
54	Economically inactive	3360	3612.62	**0.93**	**0.90-0.96**	3912.30	**0.86**	**0.83-0.89**

Table S14. Unadjusted and tobacco and alcohol adjusted SIRs for rectal cancer among 7,454,847 women in the Nordic countries, by occupation. Follow up 1961–2005.

			Unadjusted			Adjusted		
No	Occupational category	Obs	Exp	SIR	95% CI	Exp	SIR	95% CI
1	Technical workers, etc	215	199.59	1.08	0.94-1.23	195.39	1.10	0.96-1.26
2	Laboratory assistants	80	94.65	0.85	0.67-1.05	92.32	0.87	0.69-1.08
3	Physicians	53	53.14	1.00	0.75-1.30	52.85	1.00	0.75-1.31
4	Dentists	59	49.01	1.20	0.92-1.55	49.97	1.18	0.90-1.52
5	Nurses	745	808.19	**0.92**	**0.86-0.99**	764.61	0.97	0.91-1.05
6	Assistant nurses	973	1001.45	0.97	0.91-1.03	976.68	1.00	0.93-1.06
7	"Other health workers"	580	562.33	1.03	0.95-1.12	557.72	1.04	0.96-1.13
8	Teachers	1448	1515.87	0.96	0.91-1.01	1446.39	1.00	0.95-1.05
9	Religious workers etc	423	449.80	0.94	0.85-1.03	436.30	0.97	0.88-1.07
10	Artistic workers	103	106.72	0.97	0.79-1.17	110.01	0.94	0.76-1.14
11	Journalists	40	43.72	0.91	0.65-1.25	44.15	0.91	0.65-1.23
12	Administrators	326	327.15	1.00	0.89-1.11	329.63	0.99	0.88-1.10
13	Clerical workers	4908	4556.87	**1.08**	**1.05-1.11**	4686.51	**1.05**	**1.02-1.08**
14	Sales agents	599	549.64	**1.09**	**1.00-1.18**	559.61	1.07	0.99-1.16
15	Shop workers	3342	3160.02	**1.06**	**1.02-1.09**	3170.63	**1.05**	**1.02-1.09**
16	Farmers	1274	1479.40	**0.86**	**0.81-0.91**	1320.80	0.96	0.91-1.02
17	Gardeners	1441	1578.27	**0.91**	**0.87-0.96**	1481.83	0.97	0.92-1.02
18	Fishermen	7	9.69	0.72	0.29-1.49	9.60	0.73	0.29-1.50
19	Forestry workers	14	17.23	0.81	0.44-1.36	17.26	0.81	0.44-1.36
22	Transport workers	49	45.94	1.07	0.79-1.41	46.98	1.04	0.77-1.38
23	Drivers	85	86.19	0.99	0.79-1.22	88.39	0.96	0.77-1.19
24	Postal workers	701	697.43	1.01	0.93-1.08	691.69	1.01	0.94-1.09
25	Textile workers	1663	1575.06	**1.06**	**1.01-1.11**	1593.06	1.04	0.99-1.10
26	Shoe and leather workers	141	131.83	1.07	0.90-1.26	133.43	1.06	0.89-1.25
27	Smelting workers	20	28.80	0.69	0.42-1.07	29.50	0.68	0.41-1.05
28	Mechanics	230	243.40	0.94	0.83-1.08	252.52	0.91	0.80-1.04
30	Welders	14	9.47	1.48	0.81-2.48	9.71	1.44	0.79-2.42
31	Electrical workers	195	184.77	1.06	0.91-1.21	190.38	1.02	0.89-1.18
32	Wood workers	122	128.76	0.95	0.79-1.13	127.03	0.96	0.80-1.15
33	Painters	18	18.20	0.99	0.59-1.56	18.48	0.97	0.58-1.54
34	"Other construction workers"	36	34.44	1.05	0.73-1.45	38.28	0.94	0.66-1.30
36	Printers	164	145.45	1.13	0.96-1.31	153.14	1.07	0.91-1.25
37	Chemical process workers	133	125.72	1.06	0.89-1.25	127.51	1.04	0.87-1.24
38	Food workers	598	586.19	1.02	0.94-1.11	593.46	1.01	0.93-1.09
39	Beverage workers	19	32.95	0.58	0.35-0.90	34.21	0.56	0.33-0.87
40	Tobacco workers	58	36.44	**1.59**	**1.21-2.06**	38.86	**1.49**	**1.13-1.93**
41	Glass makers etc	290	289.87	1.00	0.89-1.12	299.29	0.97	0.86-1.09
42	Packers	480	456.36	1.05	0.96-1.15	467.13	1.03	0.94-1.12
43	Engine operators	28	32.96	0.85	0.56-1.23	34.52	0.81	0.54-1.17
44	Public safety workers	42	36.04	1.17	0.84-1.58	35.88	1.17	0.84-1.58
45	Cooks and stewards	651	618.68	1.05	0.97-1.14	642.38	1.01	0.94-1.09
46	Domestic assistants	1567	1584.32	0.99	0.94-1.04	1541.76	1.02	0.97-1.07
47	Waiters	699	645.49	**1.08**	**1.00-1.17**	702.86	0.99	0.92-1.07
48	Building caretakers	2790	2812.29	0.99	0.96-1.03	2908.89	0.96	0.92-1.00
50	Hairdressers	256	253.98	1.01	0.89-1.14	264.55	0.97	0.85-1.09
51	Launderers	347	354.24	0.98	0.88-1.09	364.96	0.95	0.85-1.06
53	"Other workers"	1185	1116.28	**1.06**	**1.00-1.12**	1146.64	1.03	0.98-1.09
54	Economically inactive	30344	30679.88	0.99	0.98-1.00	30678.37	0.99	0.98-1.00
